# Genetic trends in CIMMYT’s tropical maize breeding pipelines

**DOI:** 10.1038/s41598-022-24536-4

**Published:** 2022-11-22

**Authors:** Boddupalli M. Prasanna, Juan Burgueño, Yoseph Beyene, Dan Makumbi, Godfrey Asea, Vincent Woyengo, Amsal Tarekegne, Cosmos Magorokosho, Dagne Wegary, Thokozile Ndhlela, Mainassara Zaman-Allah, Prince M. Matova, Kabamba Mwansa, Kingstone Mashingaidze, Pedro Fato, Adefris Teklewold, B. S. Vivek, P. H. Zaidi, M. T. Vinayan, Nagesh Patne, Sujay Rakshit, Ramesh Kumar, S. L. Jat, S. B. Singh, Prakash H. Kuchanur, H. C. Lohithaswa, N. K. Singh, K. B. Koirala, Salahuddin Ahmed, Felix San Vicente, Thanda Dhliwayo, Jill E. Cairns

**Affiliations:** 1 International Maize and Wheat Improvement Center (CIMMYT), ICRAF Campus, UN Avenue, Gigiri, P.O. Box 1041, Nairobi, 00621 Kenya; 2 CIMMYT, El Batan, Texcoco, Mexico D.F Mexico; 3 National Crops Resources Research Institute (NaCRRI), National Agricultural Research Organization, P.O. Box 7084, Kampala, Uganda; 4 Kenya Agricultural and Livestock Research Organization (KALRO), P.O. Box 169, Kakamega, 50100 Kenya; 5 CIMMYT, P.O. Box MP163, Harare, Zimbabwe; 6 Department of Research and Specialist Services (DR&SS), Crop Breeding Institute, 5th Street Extension, Harare, Zimbabwe; 7 Zambia Agricultural Research Institute (ZARI), Lusaka, Zambia; 8 Agricultural Research Council (ARC)-Grain Crops Institute, Potchefstroom, South Africa; 9 Agricultural Research Institute of Mozambique (IIAM), Maputo, Mozambique; 10 CIMMYT, Addis Ababa, Ethiopia; 11 CIMMYT, ICRISAT Campus, Patancheru, Greater Hyderabad, Telangana India; 12 ICAR-Indian Institute of Maize Research (IIMR), Ludhiana, Punjab India; 13 University of Agricultural Sciences (UAS), Raichur College of Agriculture, Bheemarayanagudi, Yadagiri, Karnataka India; 14 University of Agricultural Sciences (UAS), Bangalore, Karnataka India; 15 G.B. Pant, University of Agriculture and Technology, Pantnagar, Uttarakhand India; 16 Nepal Agricultural Research Council (NARC), Kathmandu, Nepal; 17 Bangladesh Wheat and Maize Research Institute (BWMRI), Dinajpur, Bangladesh; 18 Present Address: Zamseed, Lusaka, Zambia; 19 Present Address: Mukushi Seeds (Pvt) Ltd, Harare, Zimbabwe

**Keywords:** Genetics, Plant sciences

## Abstract

Fostering a culture of continuous improvement through regular monitoring of genetic trends in breeding pipelines is essential to improve efficiency and increase accountability. This is the first global study to estimate genetic trends across the International Maize and Wheat Improvement Center (CIMMYT) tropical maize breeding pipelines in eastern and southern Africa (ESA), South Asia, and Latin America over the past decade. Data from a total of 4152 advanced breeding trials and 34,813 entries, conducted at 1331 locations in 28 countries globally, were used for this study. Genetic trends for grain yield reached up to 138 kg ha^−1^ yr^−1^ in ESA, 118 kg ha^−1^ yr^−1^ South Asia and 143 kg ha^−1^ yr^−1^ in Latin America. Genetic trend was, in part, related to the extent of deployment of new breeding tools in each pipeline, strength of an extensive phenotyping network, and funding stability. Over the past decade, CIMMYT’s breeding pipelines have significantly evolved, incorporating new tools/technologies to increase selection accuracy and intensity, while reducing cycle time. The first pipeline, Eastern Africa Product Profile 1a (EA-PP1a), to implement marker-assisted forward-breeding for resistance to key diseases, coupled with rapid-cycle genomic selection for drought, recorded a genetic trend of 2.46% per year highlighting the potential for deploying new tools/technologies to increase genetic gain.

## Introduction

One-third of the global area under maize production is in tropical areas of lower and lower-middle income countries^1^. Over the past 25 years, maize production has almost doubled, where about half of the increase came from an expansion in the area under maize production and the other half due to increased yields^1^. However, this production figure masks significant variation between countries and regions. While maize yields have increased globally by over 2.0 t ha^-1^ in the past 25 years, there is significant disparity in the rate of yield growth observed among the high- and low-income countries^2^. A major cause for concern is the decline or stagnation in the maize yields of many lower- and lower-middle income countries over the last two decades^3^. In sub-Saharan Africa (SSA) production gains over the last 50 years are largely associated with an increase in area rather than yields^4^. At current yield levels, the expansion of maize area is the only way to meet future food security needs^5^, yet area-based expansion for increased crop production is not sustainable and is also a major driver of biodiversity loss^6^. Increased production based on improved genetics and agronomy is essential to sustainably meet the needs of future generation.

Adding to the problems of maize production in already stress-prone environments, the frequency of climate-induced stresses is increasing in the tropical rainfed regions where maize is predominantly grown in SSA, Asia and Latin America. In South Asia, heat stress currently affects over 50% of the maize area for at least two months of the year^7^. Recent modelling studies suggest that anticipated impacts of climate change on maize production will be more pronounced than previously projected, particularly in major maize growing countries, including Mexico^8^. Although increasing maize productivity and minimizing the impacts of increasing climate variability will require intensive multi-disciplinary efforts, crop genetic improvement has historically played an important role in raising cereal productivity and reducing the impacts of climate variability on food security^9^.

Over the past 70 years in the US Corn Belt, breeding has increased maize yields by an estimated 0.10 t ha^−1^ yr^−1^^10^. This gain was initially driven by development of heterotic groups^11^, the use of reciprocal recurrent selection^12^, or doubled haploid technologies^13^, expansion of field-testing networks, improved phenotyping, data management and analytical protocols^14^. Since the start of the twenty-first century, genetic gains have been largely attributed to the use of molecular technologies to predict and select families and individuals prior to field testing^15^. Agronomic improvements have also made a significant contribution to yield gains in favourable environments^16^. Unlike the situation in the Global North, public sector maize breeding, especially through the collaboration between CGIAR and National Agricultural Research System (NARS) remains the primary source of genetic innovations and impactful products for many small- and medium-enterprise (SME) seed companies in the Global South^17,18^. Although the application of innovative breeding tools in public sector maize breeding in the Global South is still constrained, in part, by sustainable funding^19^, there has been a significant shift towards modernizing the breeding programs. Increasing accountability and tracking changes through a continuous improvement plan are essential steps in the reorganisation and modernization of breeding programs^20^.

Estimating genetic gain within a breeding program provides an opportunity to monitor breeding efficiency and effectiveness. Genetic gain estimation in maize is routinely implemented by the large multinational seed companies^21^. However, to date, there are very few estimates of genetic gains in public sector breeding programs^19^. “Era studies”, whereby varieties released in different years are evaluated in common trials, provide the most unbiased estimates of genetic gain because they avoid differences in agronomic management or climate variability which can confound the genetic trend^23^; however, these studies require significant investment of resources. The first estimation of genetic gain in CIMMYT’s eastern and southern Africa (ESA) hybrid maize breeding program used 17,018 rows (excluding seed multiplication) ^24^ which is equivalent to almost two-thirds of the number of trials used at Stage 4 (regional on-station testing) in one product pipeline in southern Africa. This budget could be more effectively allocated towards direct breeding costs to improve genetic gain rather than monitoring through era studies. Sourcing seed and re-assembling old hybrids for era studies is also a logistical challenge. Furthermore, while era studies provide a more accurate estimation of genetic gain, they do not allow real time monitoring, timely diagnosis, and correction of problems (for example, whether a breeding strategy is optimally working or is too expensive).

The primary objectives of this study were (a) to estimate the genetic trends in grain yield in 11 tropical maize breeding pipelines of CIMMYT across SSA, South Asia, and Latin America using the data from historical/advanced trials over the last 10 years to provide a baseline for future investments in tropical maize breeding; and (b) to suggest key elements for routine implementation of genetic trend estimation and improvement of tropical maize*.*

## Results

### Grain yield and repeatability

**Figure 1:**
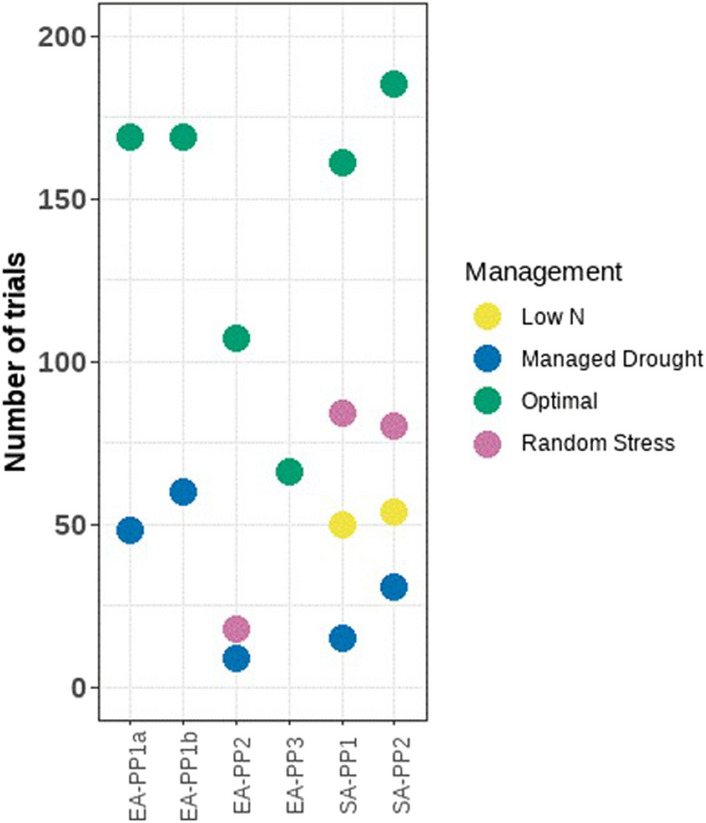
Distribution of trials under different management practices in six product profiles in eastern and southern Africa.

**Figure 2:**
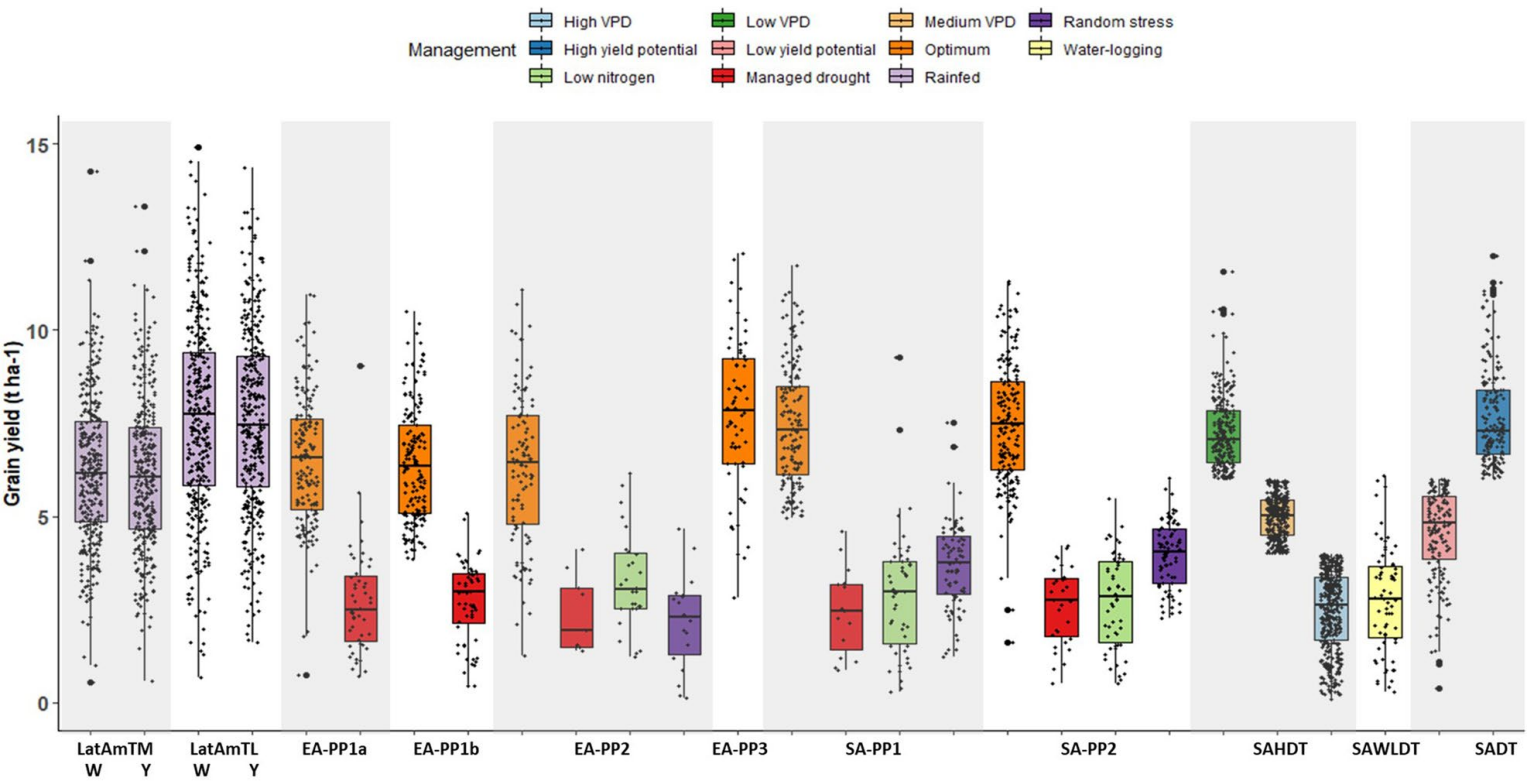
Average grain yield of individual trials in various product profiles by abiotic stress management.

**Figure 3:**
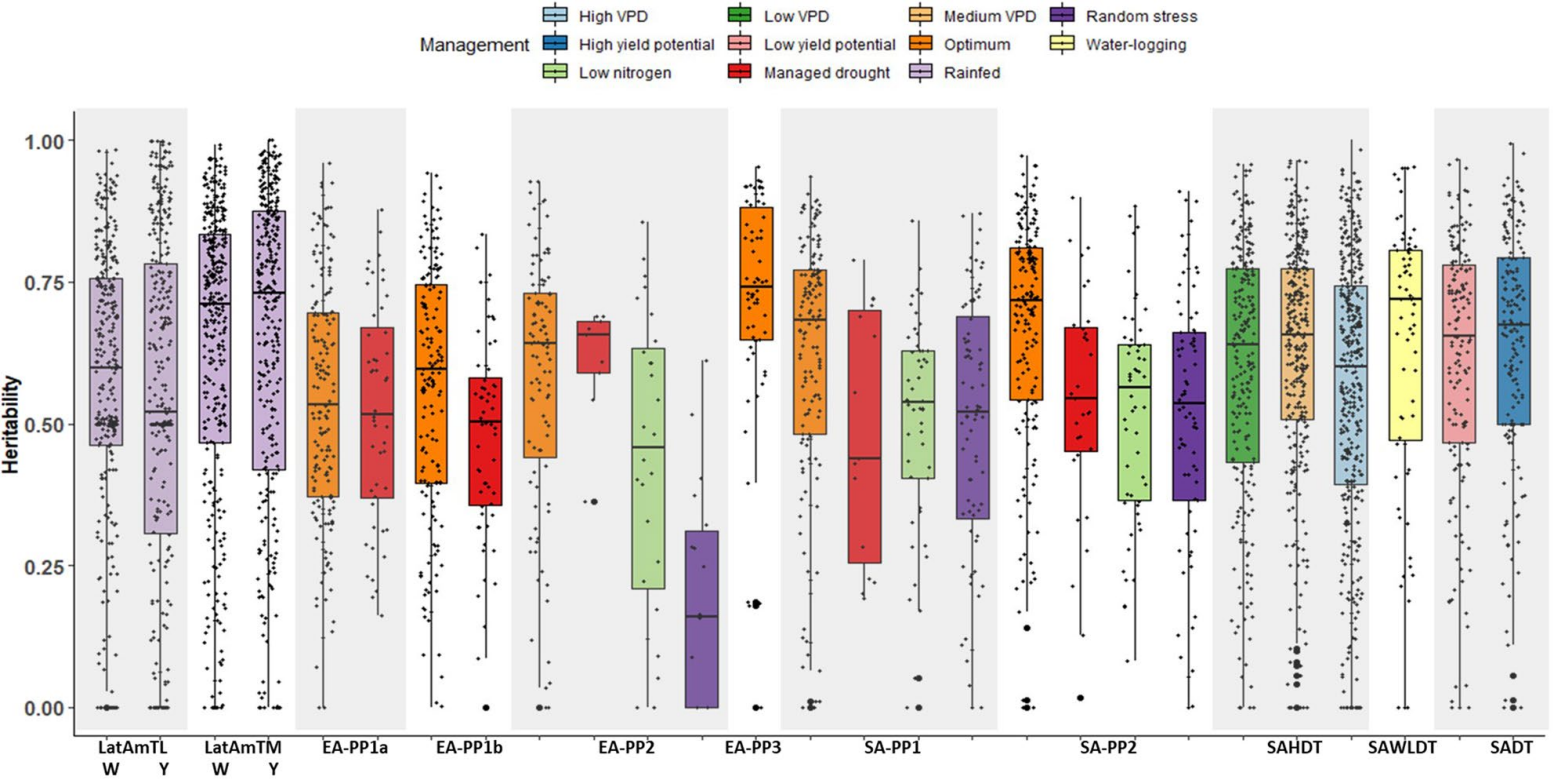
Repeatability of individual trials in various product profiles by abiotic stress management.

The number of trials for each environment ranged from 60 (EA-PP3) to 874 (SAHDT). In ESA, an array of abiotic stress environments was used within each breeding pipeline, with over 50% of trials under optimal conditions (Fig. 1). Yield potential was the highest in the LatAmTL breeding pipeline. In Asia, the lowest average yields (< 3.0 t ha^−1^) were recorded in trials screened under high VPD stress followed by trials evaluated under managed waterlogging stress with more than half of the trials recording grain yield less than 2.5 t ha^−1^ (Fig. 2). Overall, 11% of trials were removed from the analysis due to repeatability less than 0.2 (Fig. 3). Trials with low repeatability were more prevalent under random stress. In EA-PP2, 58% of random stress trials were removed due to low repeatability, and in SA-PP1 and SA-PP2 14% of random stress trials were removed due to low repeatability. In South Asia and Latin America, the number of trials removed from the analysis ranged from 4% (SADT, rainfed high production) to 13% (SAHDT, high vapour pressure deficit (VPD)), and 11% (LatAmTL White) to 21% (LatAmTL Yellow).

### Genetic trends

**Figure 4:**
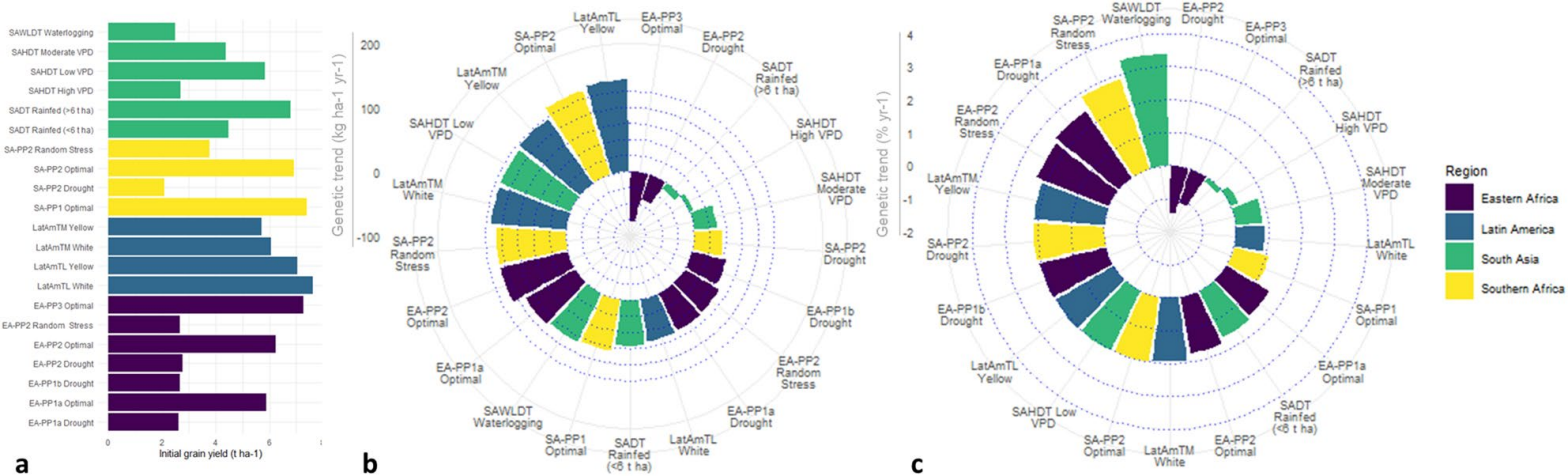
(**a**) Average grain yield in each of the breeding pipelines by individual management practices in the first year; genetic trend across product profiles presented in (**b**) kg ha^−1^ yr^−1^ and (**c**) % yr^−1^. Non-significant trends are not presented. Abbreviations: Drt, drought; HYP, high yield potential; LYP, low yield potential; Med, medium; Opt, optimum; RS, random stress; VPD, vapour pressure deficit; W, white; Y, yellow.

Linearized genetic trend for grain yield in each pipeline is presented in Fig. 4 and in Tables S7 and S9. Under optimal management, the linearized genetic trend for grain yield was, in general, high in the ESA breeding pipelines, ranging from 80 kg ha^−1^ yr^−1^ (SA-PP1) to 138 kg ha^−1^ yr^−1^ (SA-PP2). However, in EA-PP3, there was a negative genetic trend (77 kg ha^−1^ yr^−1^) for grain yield. Under managed drought stress, genetic trend ranged from −40 kg ha^−1^ yr^−1^ (EA-PP2) to 64 kg ha^−1^ yr^−1^ (EA-PP1a). There was no significant trend in grain yield over time under managed low N stress in all three product pipelines (EA-PP2, SA-PP1 and SA-PP2) that have low N tolerance in the product profile.

In Latin America, estimated genetic trend for grain yield was over 100 kg ha^−1^ yr^−1^ under rainfed conditions in both breeding pipelines, except in case of LatAmTM White which recorded 66 kg ha^−1^ yr^−1^ grain yield. Estimated genetic trend for grain yield in the LatAmTM Yellow and LatAmTL Yellow breeding pipelines was 143 kg ha^−1^ yr^−1^ and 122 kg ha^−1^ yr^−1^, respectively, under rainfed conditions. In the LatAmTM White and LatAmTL White pipelines, estimated genetic trend for grain yield was 66 kg ha^−1^ yr^−1^ and 117 kg ha^−1^ yr^−1^, respectively.

In South Asia, the estimated genetic trend for grain yield ranged from 6 kg ha^−1^ yr^−1^ under high VPD in the SAHDT pipeline to 118 kg ha^−1^ yr^−1^ under low VPD in the same pipeline. There was a small, but significant, negative genetic trend for grain yield in SADT under rainfed (high yielding) conditions. The highest genetic trend in grain yield in South Asia was recorded under managed waterlogging stress in the SAWLDT pipeline at 3.39% per year (84 kg ha^−1^ yr^−1^).

## Discussion

Previously public sector breeding programs were mostly assessed by the number of varieties released^20^; however, varietal releases alone do not reflect the efficiency of a breeding program nor the impact of a breeding pipeline. This paper presents an overview of genetic progress in CIMMYT’s maize breeding pipelines using historical data from the past decade and is the first-time genetic trends in CIMMYT’s tropical maize breeding pipelines in South Asia and Latin America (mid-altitude) are documented. When expressed as a percentage, the highest genetic trend (3.39%) was observed in the SAWLDT pipeline for grain yield under managed waterlogging stress. A similar trend was also observed in the SAHDT pipeline (targeting heat + drought stress tolerance) for trials evaluated under low VPD stress. As nascent product pipelines, with no prior genetic improvement for waterlogging tolerance in Asia prior to 2008 and heat stress before 2010, gains can be expected to be initially high. In addition, the number of trials used for analysis under waterlogging stress were comparatively low (67), primarily due to the limitations on trials that could be handled under this pipeline due to the complexity involved in imposition of the stress, besides limited funding. In this study, trials with low repeatability were removed from the estimation of genetic trend. It is possible that the inclusion of trials with repeatability (< 0.2) could change the estimates of genetic trend. However, trials with low repeatability do not form part of the stage-gate advancement process and are removed from the combined analysis to identify elite hybrids to advance to on-farm testing (Stage 5).

Cordova et al. (2007)^25^ estimated the genetic trend within the LatAmTL White breeding pipeline at 279 kg ha^−1^ yr^−1^ during 1994–2002. In the present study, the genetic trend for this pipeline (122 kg ha^−1^ yr^−1^) is significantly lower than that estimated by Cordova et al. (2007) ^25^. One of the major factors could be the significant reduction in funding for the Latin American maize breeding, including the closure of the mid-altitude breeding program in 2004 until 2011 when the Mexican Government started investing in maize improvement through CIMMYT and partners.

In general, genetic gain under optimal conditions in ESA was greater than 1% per year, although EA-PP3 was an exception to this trend. Kebede et al. (2019)^26^ reported genetic gain in grain yield of 62.26 kg ha^−1^ yr^−1^ (1.24% yr^−1^) between 1973 to 2012 in the same mega-environment; however, this era study covered old open-pollinated varieties (OPVs) to new hybrids; thus, significantly higher trends could be expected. Over the past decade, funding for the highland maize breeding pipeline (EA-PP3) has remained highly unstable. Until recently, the main source populations were lines developed from Ecuador-573, Kitale-SYN and Pool-9A with limited genetic pool. Nevertheless, in Ethiopia, there has been a sustained increase in maize yield at the country level in the last one decade due to various factors, including replacement of old and obsolete hybrids with improved varieties^27^.

Genetic progress in grain yield under managed drought stress in the ESA breeding pipelines was variable. However, yield trends in EA-PP1a, EA-PP1b and SA-PP2 were higher than those reported by Masuka et al. (2017) for the previous decade (2000 to 2010). In EA-PP2 though there was a negative trend for grain yield under drought stress, while no significant change in grain yield under drought stress was recorded in SA-PP1. These results may be a function of the number of sites available for managed drought stress screening, with only one location used in eastern Africa and two locations in southern Africa (there were previously four in this region but two of these sites could not be sustained since 2015 due to budget constraints). Genomic selection for grain yield under drought is now being implemented in ESA breeding pipelines where drought is a priority trait, allowing high selection accuracy under drought where repeatability is low compared to optimum trials. EA-PP1a was the first breeding pipeline to implement genomic selection for grain yield under drought stress^28^. The genetic trend for grain yield under drought stress in this pipeline was 2.46% per year (64 kg ha^−1^ yr^−1^). Although the present study did not allow partitioning of the gains and relate them to specific breeding tools/technologies used, when expressed as a percentage the genetic trend for grain yield was two-fold greater in EA-PP1a than in EA-PP1b (2.06% per year, 54 kg ha^−1^ yr^−1^) and SA-PP2 (2.13% per year, 45 kg ha^−1^ yr^−1^). This result is promising as other breeding pipelines, especially in Africa and Asia, are now mainstreaming genomic selection for improving grain yield under drought stress. In SSA, average fertilizer rate per hectare is estimated at 17.9 kg N ha^−1^^29^. For many farmers, the yield response to inorganic fertilizer application is often too low and variable to be profitable^30^, thus increasing the return on investment to fertilizer use through increased yields under low input conditions is essential. Masuka et al. (2017)^21^ previously found genetic gain for grain yield (20 kg ha^−1^ yr^−1^) was the lowest under low N stress but hypothesized increased investment in low N field screening capacity could potentially increase future gains. Surprisingly there was no significant genetic trend for grain yield under low N in breeding pipelines where this is considered as a key trait (EA-PP2, SA-PP1, and SA-PP2). Initial investment into breeding for low N focused on the creation of a separate low N breeding pipeline from 2009 to 2016 and this may, in part, account for a slower rate of genetic gain. Phenotyping under low N stress is expensive due to the requirement of growing a depletion crop to maintain plot uniformity; site uniformity is also hard to maintain^28^. Ertiro et al. (2020)^29^ previously showed grain yield under low N stress to be amenable to genomic selection; results from the present study suggest this may be a more powerful tool to increase the genetic trend for grain yield under low N within a fixed budget rather than through phenotypic selection alone.

Compared to CIMMYT’s other maize breeding hubs in Latin America and ESA, the Asia maize breeding program was relatively new, with its establishment in the 1980s in Thailand, from where it was moved to Nepal in 2002, and finally to India in 2003. A regional maize breeding hub was established in Hyderabad, India, in 2008. Until the mid-2000s public maize breeding programs in South Asia focused primarily on high yield potential and disease resistance. In Thailand breeding targets included high yield potential, lodging tolerance, disease resistance (*Turcicum* Leaf Blight and Downy Mildew), and nutritional quality (quality protein maize), and later Gray Leaf Spot (GLS) and Banded Leaf and Sheath Blight (BLSB). Breeding pipeline for drought tolerance in Asia was initiated in 2008, with the introgression of drought tolerant (white and yellow) lines from Mexico, and white lines from Africa since 2010. The introgression of drought tolerance from white donor lines into a yellow recipient to derive viable yellow lines eliminates a large number of segregating inbred lines based on colour alone. Thus, large population sizes needed to be screened to be able to derive viable yellow drought tolerant lines, thereby reducing selection intensity for key traits. Interestingly, the genetic trend for grain yield under drought in the SADT pipeline (71 kg ha^−1^ yr^−1^) is higher than previously estimated 32.5 to 55 kg ha^−1^ yr^−1^ by Masuka et al. (2017)^24^ under drought stress in ESA. The breeding pipeline SAHDT targets rainfed maize growing environments with the crop experiencing a combination of drought and heat. While heat stress screening was started by CIMMYT in the mid-1980s in Obregon, Mexico, in Asia, breeding for heat tolerance was initiated only in 2012, in partnership with national maize programs and seed companies in Bangladesh, India, Nepal and Pakistan. Genetic gain estimates for this increasingly important trait varied from 0.24% (high VPD), 0.82% (medium VPD) and 2.02% (low VPD). Substantial progress has been made through this pipeline, with successful release and commercialization of heat tolerant maize hybrids across partner countries in South Asia.

Emerging threats over the past decade, including Maize Lethal Necrosis (MLN) in eastern Africa^17^, Fall Armyworm (FAW) in SSA and in Asia^33^, and Tar Spot Complex (TSC) in Latin America^34^, have resulted in the addition of new target traits to CIMMYT’s tropical maize product profiles. These new threats also resulted in significant shifts in the base genetics, and reduced germplasm exchange (mainly due to the risk of transfer of MLN-contaminated seed) among breeding hubs as germplasm movement was restricted^35^. Key lines in the ESA breeding pipelines, which are widely used in commercial products in eastern Africa, were highly susceptible to MLN^35^ and required infusion of new genetic variation from other regions. This may have, in part, led to slowing the genetic gain in some breeding pipelines. However, lessons learned in the response to these recent threats will play an important role in reducing the reaction time to future threats. The ability to mobilise relevant germplasm across international borders, and capacity to quickly utilise new breeding tools with a clear vision to deployment are vital. For example, within months of MLN outbreak in Kenya thousands of maize inbred lines from a diverse range of breeding programs worldwide were screened by CIMMYT under artificial inoculation for MLN resistance, and two donor lines identified. Linkage and association mapping approaches were used to identify and subsequently validate genomic regions associated with MLN resistance. A major-effect, recessively inherited QTL (qMLN06_157) on chromosome 6 was identified, accounting for 55–70% of the phenotypic variation for traits related to MLN resistance. Marker-assisted backcrossing (MABC) was used to provide an intermediate step to maintain yields by quickly incorporating six SNPs associated with MLN tolerance into key inbred lines used in commercial products and advanced (pre-release) hybrids. Within 2.5 years, 24 key inbred lines were converted to MLN resistance using MABC^36^. In parallel, forward breeding was used to enrich populations for MLN resistance prior to field phenotyping in EA-PP1. Lines developed using this approach have now entered Stage 4 (regional trial) testing^17,37^. The fast, coordinated adaptation of breeding pipelines, identification of donor lines with favourable QTLs/SNPs, and the release of first set of hybrids with MLN tolerance within three years after disease outbreak in eastern Africa^35^ were major features of this rapid response to MLN. Similarly, first-generation hybrids with native genetic resistance to FAW were identified and licensed to NARS partners within four years of the pest outbreak^33^, and genomic analysis of native genetic resistance to FAW was also undertaken^38^.

The major aim of this study was to establish a baseline for all CIMMYT breeding pipelines rather than compare genetic trends between regions which face diverse challenges. In our study, comparison between regions is of limited value due to the varied age of breeding programs, different levels of investments, and diverse requirements of tropical maize pipelines including types of products. Since 2006, there has been significant and continuous investment in maize breeding in ESA, except for EA-PP3^39^. The level of funding for maize breeding in both Latin America and South Asia was however much lower, with greater fluctuations, compared to ESA. Over the past decade there has been a significant effort to modernize CIMMYT maize breeding programs. Different funding levels are also reflected in application of tools/technologies to increase the rate of genetic gain (Table 1).

**Table 1:** Implementation of key breeding tools/processes to improve genetic gains in CIMMYT’s tropical maize breeding pipelines.

	Eastern Africa			Southern Africa		South Asia			Latin America	
	EA-PP1	EA-PP2	EA-PP3	SA-PP1	SA-PP2	SAWLDT	SAHDT	SADT	LatAmTL	LatAmTM
Product profiles refined and aligned across CIMMYT, NARS and SMEs	2019	2019	2019	2019	2019	2019	2019	2019	2019	2019
Implementation of sparse phenotyping	2021	2021	2022	2021	2022	2022	2021	2022	–	–
Use of selection index at early stages of breeding for selecting parents for recycling	2021	2021	2022	2022	2022	2022	2022	2022	2021	2021
Current breeding cycle time (in years)	4 (recycling parents after Stage 2 testing)	4 (recycling parents after Stage 2 testing)	5 (recycling parents after Stage 3 testing)	4 (recycling parents after Stage 2 testing)	4 (recycling parents after Stage 2 testing)	5 years (recycling parents after Stage 3 testing)	4.5 years (recycling parents after Stage 2 testing)	5 years (recycling parents after Stage 3 testing)	4 (recycling parents after Stage 2 testing)	4 (recycling parents after Stage 2 testing)
Level of integration of DH technology in breeding pipelines	80%	70%	30%	60%	60%	50%	80%	10%	50%	50%
Rapid generation advancement through three-season nurseries per year	2021: 2.5 seasons/year; From 2022: 3 seasons/year (after the seed dryer installation)	2021: 2.5 seasons/year; From 2022: 3 seasons/year (after the seed dryer installation)	2021: 2 seasons/year	2021: 2.5 seasons/year; From 2022: 3 seasons/year (after the seed dryer installation)	2021: 2.5 seasons/year; From 2022: 3 seasons/year (after the seed dryer installation)	2021: 2 seasons/year	2021: 2 seasons/year	2021: 2 seasons/year	2.5 seasons/year	2.5 season/year
Marker-assisted forward breeding	MSV; MLN	MSV; MLN	MLN	MSV	MSV	Initiated for TLB in 2021	Not implemented	Initiated for TLB in 2021	TSC	PVA; MSV
Genomic prediction (Test half-Predict half)	Since 2017 (all Stage 1 genotyped with mid-density markers); selection of individuals for the next stage of testing based on GEBVs and GBLUPs	Since 2017 (all Stage 1 genotyped with mid-density markers); selection of individuals for the next stage of testing based on GEBVs and GBLUPs	All Stage 1 genotyped with mid-density markers in 2021, and GEBVs estimated for selection of lines for the next stage of testing	All Stage 1 genotyped with mid-density markers in 2021, and GEBVs estimated for selection of lines for the next stage of testing	Not started	Not started	Being implemented since 2019	Not started	From 2015 to 2020, (all Stage 1 genotyped with mid to high-density markers); selection of individuals for the next stage of testing based on GEBVs and GBLUPs)	Proof-of-Concept (2019)
Rapid cycle genomic selection (RCGS)	Being implemented	Being initiated in 2022	Not started	Being initiated in 2022	Being initiated in 2022	Implemented for two multi parent populations during 2010–2013	Implemented for six multi-parent populations during 2014–2017	Implemented 2 cycles for six populations during 2010–2013	Tested from 2010 to 2015; Being initiated in 2022	Being initiated in 2022
Molecular markers-based QA/QC in CGIAR, NARS & selected SMEs	Yes	Yes	Yes	Yes	Yes	Yes	Yes	Yes	Yes	Yes
Implementing high-throughput field-based phenotyping	Being initiated in 2022 (PH, EH)	Being initiated in 2022 (PH, EH)	Being initiated in 2022 (PH, EH)	Being implemented (PH, EH)	Being implemented (PH, EH)	Implemented till Dec, 2021 (PH, AD)	Implemented till Dec, 2021 (PH, AD)	Not started	UAVs tested for drought stress	UAVs tested for drought stress
Joint CGIAR-NARS annual stage-gate advancement meetings	Yes	Yes	Yes	Yes	Yes	To be implemented	To be implemented	To be implemented	Yes	Yes

Abbreviations: AD, Anthesis date; DH, Doubled haploid; EH, Ear height; GBLUP, Genomic best linear unbiased prediction; GEBV, Genomic estimated breeding value; MLN, Maize Lethal Necrosis; MSV, Maize Streak Virus, PH, Plant height; PVA, Provitamin A; TLB, Turcicum Leaf Blight; TSC, Tar Spot Complex; UAV, Unmanned aerial vehicle.

Doubled haploid technology, forward breeding for key diseases, and genomic selection for complex traits such as drought tolerance are now being deployed in over half of CIMMYT’s breeding pipelines (Table 1). Reducing cycle time is the most efficient method of increasing genetic gain^37^. Initially, increased use of off-season nurseries allowed breeding programs to move to two seasons per year. Recent installation of seed driers in key breeding hubs will allow three seasons per year nurseries to be implemented. The breeding cycle time is now between 4 and 5 years across pipelines, a reduction of up to two years over the past decade. Forward breeding is a simple form of population enrichment using markers tightly linked to genomic regions of high importance, thereby increasing selection intensity without increasing phenotyping requirements. Almost three-quarters of the breeding pipelines in ESA now rely primarily on forward breeding for resistance to two key diseases [e.g., MLN; Maize Streak Virus (MSV)] to select DH/inbred lines prior to field testing. Marker-assisted forward breeding for selection of favourable haplotypes for resistance to MLN and MSV resulted in a saving of almost US$ 300,000 in phenotyping expenditure during a four-year period. Refinement in genomic selection strategies coupled with a reduction in cost of securing adequate marker density has enabled mainstreaming of genomic selection as an integrated breeding method within eastern Africa^28,41,42^. The use of proximal sensors for key traits used in selection is increasing across breeding pipelines, reducing the time and cost of the measurement of routine traits and allowing money to be diverted towards generation and management of larger populations^17^. Given the time taken for the key lines developed using new tools and technologies to reach Stage 4 testing, the effects of breeding modernisation are likely to be reflected in genetic trends over the next few years. Genetic trend is a function of the replacement of both female and male parental lines. Our data does not permit decomposing the estimation of genetic trends of female and male parental lines, due to the lack of replication across years. Using an era study composed of hybrids selected by national breeding programs and seed companies for release, Masuka et al. (2017)^43^ found the replacement of parental female parent inbreds of elite hybrids was slower than that of male parents in ESA. Across pipelines, in general, there has been a reduction in the age of females with increased replacement over time (Table S2), with the exception of Southern African pipelines where the age of females used has not decreased despite a large number being replaced each year. The number of distinct males, in general, increased over time although the pattern is less clear (Table S3).

The use of historical data to estimate genetic trend can be confounded due to non-genetic trends related to increased climate variability or changes in agronomic management^22^. Trials were conducted in experimental research stations; thus, there was no change in agronomic management practices during the study period. Recent changes in climate did impact maize yields^44^. The relatively short length of the study period (up to 12 years), the use of supplemental irrigation when required, and the smaller number of trials under random stress would have limited the potential to decipher the effect of non-genetic effects related to increased climate variability. Future analyses of genetic trend within breeding pipelines presented in this study should be able to estimate the effect of non-genetic effects related to the environment on genetic trends.

**Figure 5:**
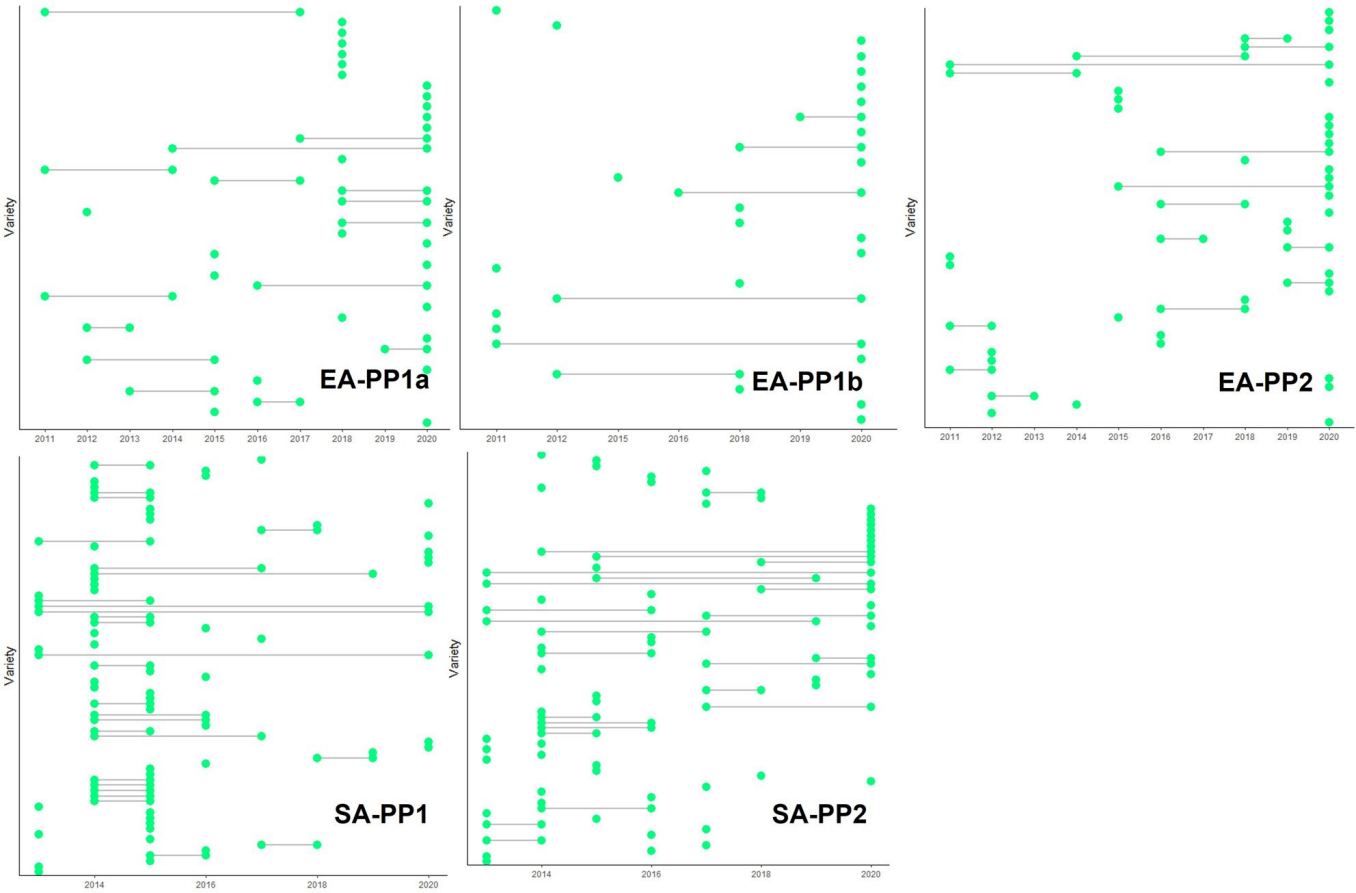
Presence of checks (both commercial and internal genetic gain) across years in the five breeding pipelines in eastern and southern Africa during the period used for genetic trend analysis.

The use of historical data to estimate genetic trend requires connectivity of checks in trials across years. This provides a degree of overlap of different cohorts and avoid confounding of estimates of genetic or breeding values with the year effect. The trials reported in this study were not originally designed with the intention of estimating genetic trend; thus, the connectivity across years and gradual check replacement strategy were not optimal. The connectivity of checks for the five breeding pipelines in ESA are presented in Fig. 5 as an example of the check strategy used by CIMMYT. In SA-PP1 where the seed sector is relatively vibrant, checks were replaced quickly to allow the comparison of elite CIMMYT hybrids with the best genetics emerging in the market. In pipelines where previously commercial varieties, representing a significant proportion of the market share, have remained on the market for over a decade the check replacement strategy has been slower. The number of consecutive years varieties were tested was generally very low with > 90% only tested in one year. The higher the number of checks used to estimate genetic trend, the power of analysis will increase by reducing the year effect. Ensuring a higher percentage of varieties are tested for at least three consecutive years would the use of statistical models that separate genetic and non-genetic trends using a two-stage analysis^45^. However, more checks have cost implications as they increase the size of each trial and reduce testing capacities. The percentage of entries which are checks (check percentage) should strike a balance between the cost of estimating genetic trend and the accuracy of the estimates. Moving forward the check replacement strategy will be harmonized across breeding pipelines to facilitate optimal genetic trend assessment and allow genetic trends to be sub-divided into genetic and non-genetic^40^.

## Conclusions

The present study provides a baseline for future maize breeding efforts in the tropical rainfed areas, especially in the low- and middle-income countries in SSA, Asia and Latin America. While genetic trends for grain yields in CIMMYT’s tropical breeding pipelines were generally slightly lower than the estimated 2.4% per year that was projected as required to meet the future needs^42^, in several pipelines the genetic trend in yield in the on-station trials were around this target. The high genetic trend for grain yield under drought stress in EA-PP1a, the first breeding pipeline to implement genomic selection in Stage 1, is highly promising; a similar approach may increase genetic gains across the pipelines. Furthermore, over the past decade, there have been significant changes in all the breeding pipelines as new breeding tools/technologies have been utilized to increase selection intensity and accuracy, besides significant exchange of elite germplasm between breeding hubs, and introgression of other sources of genetic diversity (e.g., ex-PVP temperate maize germplasm). These effects of continuous improvement are expected to manifest in terms of improved genetic trends over the next few years.

While the study showed significant progress in increasing genetic gains in the tropical maize breeding pipelines of CIMMYT, there are certainly opportunities for further improvement. Sustainable funding is needed to aggressively implement modern tools/technologies in improving selection efficiency and accuracy, and to further reduce cycle time, in addition to implementing interdisciplinary approaches for more predictive breeding^47^. Genetic gain in breeding pipelines also need to be effectively translated into gains on-farm through active replacement of old and obsolete varieties with improved genetics that can benefit the targeted farming communities, besides improved crop management/agronomy and increase in input use. All this would require stable, committed, and increased funding for tropical maize improvement in SSA, South Asia and Latin America, where the crop is largely cultivated under rainfed conditions by resource-constrained smallholders.

## Materials and methods

### Breeding pipelines and germplasm

**Figure 6:**
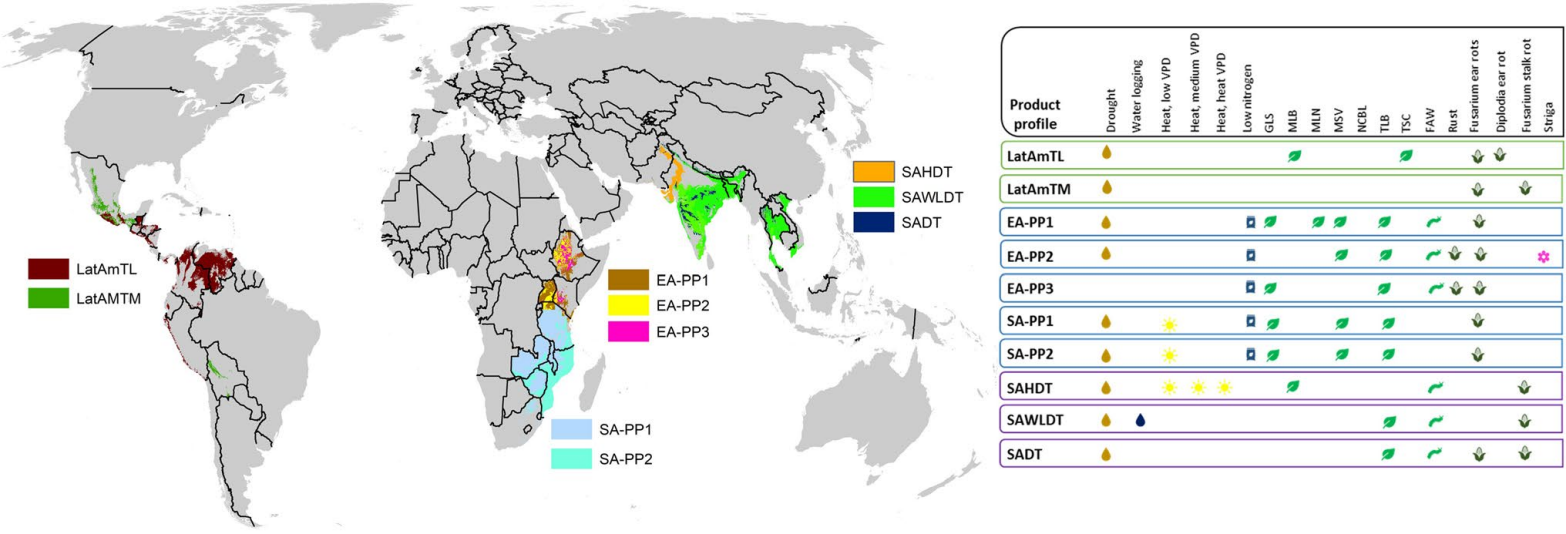
Overview of CIMMYT’s maize product profiles and the geographical area served by each of the breeding pipelines. Map created using ESRI (Environmental Systems Research Institute) ArcGIS Release 10.8.1 Redlands, CA, USA. Abbreviations: VPD, Vapour Pressure Deficit; GLS, Gray Leaf Spot; MLB, Maydis Leaf Blight; MLN, Maize Lethal Necrosis; MSV, Maize Streak Virus; TLB, Turcicum Leaf Blight; TSC, Tar Spot Complex; FAW, Fall Armyworm.

CIMMYT has three breeding pipelines in eastern Africa, two in southern Africa, three in South Asia, and two in Latin America, with varying breeding history. The geographical areas and the must-have abiotic and biotic stress traits of these breeding pipelines are captured in Fig. 6. A list of “must-have” traits is also presented in Table S4. The breeding schemes varied slightly by geography and product profile; however, carefully selected parental lines from the same heterotic group were crossed to generate source populations. The source populations were subjected to pedigree breeding or doubled haploidy (DH) to develop fixed lines. After DH lines were produced, lines were genotyped with mid-density markers and subjected to marker-assisted forward breeding for relevant traits (e.g., resistance to key diseases like Maize Streak Virus (MSV) and Maize Lethal Necrosis (MLN)) based on favourable haplotypes). A panel of ten SNPs were used for selection for MSV and MLN. SNPs were previously identified by Nair et al. (2015)^48^ and Gowda et al. (2015)^49^. Genomic selection was applied at Stage 1 by genotyping all lines with mid-density markers (1000–10,000 SNPs) using the DART genotyping platform. Selection of individuals for advancement was based on genomic estimated breeding values (GEBVs) and genomic best linear unbiased predictions (gBLUPs)^41^.Selected lines were genotyped, and then testcrossed to either a single-cross or an inbred tester. The testcrosses were then evaluated in relevant mega-environments. In some breeding pipelines, part of the F1 source populations were subjected to backcrossing to introgress specific traits (e.g., resistance to maize lethal necrosis), and the backcross families were further advanced. Lines were advanced based on their testcross performance across multiple locations from Stage 1 to Stage 3. Stage 4 trials include hybrids selected from Stage 3 trials, pre-release varieties nominated by partners i.e., both national agricultural research systems (NARS) and private seed companies, and commercial benchmark varieties, which facilitate advancement decisions. The range of environments was guided by mega-environments and trait requirements of each breeding pipeline. Stage 1–3 trials were conducted by CIMMYT in partnership with NARS, while Stage 4 and Stage 5 trials were jointly conducted by CIMMYT, NARS and seed companies. The final stage of the breeding pipeline was Stage 5 where trials were run in a regional on-farm trial network, under farmer-managed conditions. Stage gate advancement from Stage 3 onwards was based on voting by CGIAR-NARS breeding network team members. Data from both Stage 4 and Stage 5 were used in final product advancement decisions. The number of hybrids, selection intensity applied, and the number of locations per stage varied by breeding pipeline (Table S5). The number of hybrids tested at each stage varied by breeding pipelines. In Stage 1 the number of hybrids tested ranged from 600 (LaAmTL) to 3000 (EA-PP1). In Stage 2 the number of hybrids tested ranged from 250 (SADT) to 900 (EA-PP1). In Stage 3 the number of hybrids tested range from 50 (SADT) to 200 (EA-PP1). Inbred selections from Stage 3 were used for recycling and for generating new breeding sources.

### Field trials

All the genetic materials used in this study were developed at the CIMMYT maize breeding hubs, except for the commercial benchmark checks, the seed for which was sourced from the market. A total of 4152 trials were conducted at 1331 locations in 28 countries between 2009 and 2020 (Table S6). The trials were conducted by CIMMYT, in partnership with NARS and seed companies, under optimal, managed drought stress, random stress, low nitrogen stress, high vapour pressure deficit (VPD) heat stress, medium VPD heat stress, low VPD heat stress, managed waterlogging stress, wet season rainfed trials, etc. depending on the trait requirements in each pipeline. Details of the management practices are provided in Table S7. All the trials were planted following an alpha-lattice design with entries replicated two to three times. Experiments used two-row plots, with 4 to 5 m long rows, with a spacing of 0.75 m between rows and 0.25 m between plants within a row. The study was conducted in accordance with relevant institutional, national, and international guidelines and regulations.

### Statistical analysis

For the breeding pipelines in eastern and southern Africa, Stage 4 (on-station) trials were used for the estimation of genetic trend in grain yield. In case of Latin America pipelines Stage 5 (on-farm) data was used due to the larger number of years and locations compared to Stage 4 data. In Asia pipelines, data from stages 2–4 were used as the streamlining of testing strategies is more recent. The average number of consecutive years of trials used for genetic trend estimation was nine (Table S8). Following the recommendations of Excellence-in-Breeding Platform^20,50,51^, data from each breeding pipeline and trait were analysed separately. Due to unbalanced nature of the data and because this is part of an overall exercise across CGIAR crop breeding programs, a common methodology has been used that is considered best ft across a diverse array of situations in which these breeding programs operate, including perennial and annual crops, with and without molecular information, different phenotyping strategies, experimental designs, and use of checks. In the first step, individual analysis of each experiment was run to evaluate data quality considering both, the number of effective replicates (N_eff_: number of observations divided by the number of tested genotypes) and the repeatability (H^2^) were estimated. Data from trials with a N_eff_ lower than 1.25 or H^2^ lower than 0.2 were excluded from analysis. The trials were laid in α(0,1) incomplete block designs (IBD) with the exception of those in Latin America where Stage 5 trials were planted in a randomized complete block design. A standard linear mixed model for an IBD was used for individual analysis:

$${y}_{ijk}=\mu +{\delta }_{i}+\beta (\delta {)}_{j\left(i\right)}+{\alpha }_{k}+{\varepsilon }_{ijk}$$

where:

$$\begin{gathered} y_{{ijk}} :{\text{~response~variable,~grain~yield~in~t~ha}}^{{ - 1}} ,\;\mu :{\text{~overall~mean}},\;\delta _{i} :{\text{~effect~of~the~ith~replicate,}}\;\beta (\delta )_{{j\left( i \right)}} :{\text{~effect~of~the~jth~block~nested~in~the~ith~replicate,~}}\beta (\delta )_{{j\left( i \right)}} = N\left( {0,\sigma _{\beta }^{2} } \right),\; \hfill \\ \alpha _{k} :{\text{~effect~of~the~kth~genotype,~}}\alpha _{k} = N\left( {0,\sigma _{\alpha }^{2} } \right),\;\varepsilon _{{ijk}} :{\text{~experimental~error,~}}\varepsilon _{i} = N\left( {0,\sigma _{\varepsilon }^{2} } \right) \hfill \\ \end{gathered}$$

Repeatability was calculated with:

$$H^{2} = 100\frac{{\sigma _{\alpha }^{2} }}{{\sigma _{\alpha }^{2} + {\raise0.7ex\hbox{${\sigma _{\varepsilon }^{2} }$} \!\mathord{\left/ {\vphantom {{\sigma _{\varepsilon }^{2} } {N_{{eff}} }}}\right.\kern-\nulldelimiterspace} \!\lower0.7ex\hbox{${N_{{eff}} }$}}}}$$

For the randomized complete block design, block nested in replicate was removed from the model; the replicate effect represents complete-block effect. To remove environmental effects on genotype effects, a linear mixed model was fitted for combined analysis across years. Because the checks, which represent dominant commercial hybrids in the market, were the only genotypes that were replicated across years, they play an important role in the estimation of effects including the year effect. They are the comparison point between genotypes released at different years. The model included year, location, genotypes and their interaction effect plus experimental design effect. Year and genotype were fixed effects, and other effects were considered as random effects^44^:

$${y}_{ijklmn}=\mu +{\delta (\gamma )}_{i(j)}+{\beta (\gamma \delta )}_{k(ij)}+{\lambda }_{l}+{\alpha }_{m}+{\lambda \alpha }_{lm}+{\eta }_{n}+{\lambda \eta }_{ln}+{\alpha \eta }_{mn}+{\lambda \alpha \eta }_{lmn}+{\gamma (\lambda \eta )}_{p(ln)}+{\gamma \alpha (\lambda \eta )}_{im(ln)}+{\varepsilon }_{ijklmn}$$

where

$$y:~{\text{response~variable,~}}\mu :~{\text{overall~mean,~}}\delta \left( \gamma \right)_{{i\left( j \right)}} :~{\text{effect~of~the~}}ith{\text{~replicate~nested~in~the~}}jth{\text{~experiment,~}}\delta \left( \gamma \right)_{{i\left( j \right)}} = ~N\left( {0,\sigma _{\delta }^{2} } \right),~\beta \left( {\gamma \delta } \right)_{{k\left( {ij} \right)}} :~{\text{effect~of~the~~}}kth{\text{~block~nested~in~the~}}ith{\text{~replicate~in~the~}}jth{\text{~experiment,}}$$

$$\beta \left( {\gamma \delta } \right)_{{k\left( {ij} \right)}} =N\left( {0,\sigma _{\delta }^{2} } \right)$$

$$\begin{gathered} \lambda _{l} :~{\text{effect~of~the~}}lth{\text{~year,~}}\alpha _{m} :~{\text{effect~of~the~}}mth{\text{~genotype,~}} \hfill \\ \lambda \alpha _{{lm}} :~{\text{interaction~effect~of~the~}}lth{\text{~year~with~the~}}mth{\text{~genotype,~}}\lambda \alpha _{{lm}} = ~N\left( {0,\sigma _{{\lambda \alpha }}^{2} } \right),~~ \hfill \\ \eta _{n} :~{\text{effect~of~the~}}nth{\text{~location,~}}\eta _{n} = ~N\left( {0,\sigma _{\eta }^{2} } \right),~ \hfill \\ \lambda \eta _{{ln}} :{\text{interaction~effect~of~the~}}lth{\text{~year~with~the~}}nth{\text{~location, }}\lambda \eta _{{ln}} = N\left( {0,\sigma _{{\lambda \eta }}^{2} } \right),~ \hfill \\ \alpha \eta _{{mn}} :~{\text{interaction~effect~of~the~}}mth{\text{~genotype~with~the~}}nth{\text{~location,~}}\alpha \eta _{{mn}} = ~N\left( {0,\sigma _{{\alpha \eta }}^{2} } \right),~\; \hfill \\ \lambda \alpha \eta _{{lmn}} :~{\text{interaction~effect~of~the~}}lth{\text{~year~with~the~}}mth{\text{~genotype~with~the~}}nth{\text{~location,}} \hfill \\ \end{gathered}$$

$${\lambda \alpha \eta }_{lmn}=N(0,{\sigma }_{\lambda \alpha \eta }^{2})$$

$$\begin{gathered} \gamma \left( {\lambda \eta } \right)_{{j\left( {ln} \right)}} :~~{\text{effect~of~the~}}jth{\text{~experiment~nested~in~the~}}lth{\text{~year~and~}}nth{\text{~location,~}}\gamma \left( {\lambda \eta } \right)_{{j\left( {ln} \right)~}} = N\left( {0,\sigma _{\gamma }^{2} } \right),~ \hfill \\ \gamma \alpha \left( {\lambda \eta } \right)_{{jm\left( {ln} \right)}} :~{\text{interaction~effect~of~the~}}jth{\text{~experiment~with~the~}}mth{\text{~genotype,~nested~in~the~}}lth{\text{~year,~}} \hfill \\ {\text{and~}}nth{\text{~location,~}}\delta \alpha \left( {\lambda \eta } \right)_{{jm\left( {ln} \right)}} = N\left( {0,\sigma _{{\gamma \alpha }}^{2} } \right),~ \hfill \\ \varepsilon _{{ijklmn}} :~{\text{experimental~error,~}}\varepsilon _{{ijklmn}} = N\left( {0,\sigma _{\delta }^{2} } \right) \hfill \\ \end{gathered}$$

With a heterogeneous residual error by experiment,

$$\sum\nolimits_{z} { = \left[ {\begin{array}{*{20}l} {\sigma_{1}^{2} {\mathbf{I}}} \hfill & 0 \hfill & \cdots \hfill & 0 \hfill \\ 0 \hfill & {\sigma_{1}^{2} {\mathbf{I}}} \hfill & \cdots \hfill & 0 \hfill \\ \vdots \hfill & \vdots \hfill & \ddots \hfill & \vdots \hfill \\ 0 \hfill & 0 \hfill & \cdots \hfill & {\sigma_{E}^{2} {\mathbf{I}}} \hfill \\ \end{array} } \right]}$$

The adjusted means of genotypes were estimated from the combined analysis. A linear regression model of genotype adjusted means was then fitted against year of release to estimate the genetic gain. Year of release is the first year a genotype was tested in the set of experiments. From the model, the following statistics were estimated:

$${\widehat{y}}_{ip}=\alpha +\beta {x}_{i}+{\varepsilon }_{ip}$$

$$where:$$

$${\widehat{y}}_{ip}:\text{ adjusted mean of the }{\text{i}}{\text{th}}\text{ genotype released the }{\text{p}}{\text{th}}\text{ year}$$

$$\alpha :\text{ intercept}$$

$$\beta :\text{ regression coefficient, genetic gain expressed in t h}{\text{a}}^{-1} {y}^{-1}$$

$${x}_{i}:\text{ year of release of the }{\text{i}}{\text{th}}\text{ genotype}$$

$$\varepsilon _{{ip}} :{\text{~experimental~error~plus~deviation~from~the~regression~model,~}}\varepsilon _{{ip}} = N\left( {0,\sigma _{\varepsilon }^{2} } \right)$$

There are several ways to estimate genetic gain with historical data^45,52^. The methodology in this study was selected based on the structure of the data, with limited genotypes screened over consecutive years.

## Supplementary Information

Supplementary Information 1.

Supplementary Information 2.

## Data Availability

The authors have all the necessary permissions to collect and/or evaluate the experimental materials used in this study. BLUPS from all the trials are available on CIMMYT Dataverse (https://data.cimmyt.org/). Additional information is presented in the Supplementary Tables S1–S9.
