# Supplementary material for: Genetic trends in CIMMYT’s tropical maize breeding pipelines
Source: Sci Rep. 2022 Nov 22;12:20110. doi: 10.1038/s41598-022-24536-4 (PMC9684471; doi:10.1038/s41598-022-24536-4)
Supplement: Supplementary file 2 — Supplementary Information 2. [file 41598_2022_24536_MOESM2_ESM.docx]

**Supplementary Tables**

**Table S1.** “Must-have” abiotic and biotic stress traits in CIMMYT maize breeding pipelines.

|  |  | **Must-have traits** | |
| --- | --- | --- | --- |
| **Region** | **Product pipeline** | **Abiotic** | **Biotic** |
| Latin America | LatAmTL (White, Yellow) | Drought | Maydis Leaf Blight (MLB), Tar Spot Complex (TSC), Fusarium Ear Rot (FER), Diplodia Ear Rot (DER) |
|  | LatAmTM (White, Yellow) | Drought | FER, Fusarium Stalk Rot (FSR), Gray Leaf Spot (GLS) |
| Eastern Africa | EA-PP1a | Drought, low nitrogen | Gray Leaf Spot (GLS), Maize Lethal Necrosis (MLN), Maize Streak Virus (MSV), Turcicum Leaf Blight (TLB), Fall Armyworm (FAW), FER |
|  | EA-PP1b | Drought, low nitrogen | GLS, MLN, MSV, TLB, FAW, FER |
|  | EA-PP2 | Drought, low nitrogen | MSV, TLB, Common rust, FER, Striga, FAW |
|  | EA-PP3 | Low nitrogen | GLS, TLB, Common rust, FER, FAW |
| Southern Africa | SA-PP1 | Drought, low nitrogen | GLS, MSV, TLB, FER |
|  | SA-PP2 | Drought, low nitrogen | GLS, MSV, TLB, FER |
| South Asia | SAHDT | Drought, heat stress (low, medium, and high VPD) | Macrophomina Stalk Rot (MSR) |
|  | SAWLDT | Drought, waterlogging | FSR, TLB |
|  | SADT | Drought | FSR, TLB |

LatAmTL: Latin America Tropical Lowland; LatAmTM: Latin America Tropical Mid-altitude; EA-PP1a: Eastern Africa Product Profile1a; EA-PP1b: Eastern Africa Product Profile1b; EA-PP3: Eastern Africa Product Profile3; SA-PP1: Southern Africa Product Profile1; SA-PP2: Southern Africa Product Profile2; SAHDT: South Asia Heat and Drought Tolerant; SAWLDT: South Asia Waterlogging and Drought Tolerant; SADT: South Asia Drought Tolerant.

**Table S2.** The number of hybrids, testers, locations, and average selection intensity used by each breeding pipeline in 2022. Advancement decisions from Stage 3 to Stage 5 are based on a voting system involving CGIAR-NARS breeding teams; thus, the selection intensity varies between years. Selection intensity for these stages (Stages 3-5) are highlighted in italics.

| **Breeding Stage** | **Number of hybrids** | **Number of testers** | **Selection intensity (%)** | **Number of locations** | **Number of hybrids** | **Number of testers** | **Selection intensity (%)** | **Number of locations** |
| --- | --- | --- | --- | --- | --- | --- | --- | --- |
| **LaAmTL** | | | | | **LaAmMT** | | | |
| Stage 1 | 600 | 1 HGA, 1 HGB | 10-12% | 3 | 500 | 1 HGA, 1 HGB | 10-15% | 3 |
| Stage 2 | 300-360 | 3 HGA, 3 HGB | 15% | 6 | 285-360 | 3 HGA, 3 HGB | 12-15% | 6 |
| Stage 3 | 75-100 | 5 HGA, 5 HGB | *20%* | 15 | 75-90 | 5 HGA, 5 HGB | *20-25%* | 15 |
| Stage 4 | 15-20 | NA | *20%* | 20-35 | 15-20 | NA | *20%* | 20-25 |
| Stage 5 | 3-5 | NA | *(1-2)^a^* | 30-40 | 3-5 | NA | *(1-2)^a^* | 30-40 |
| **EA-PP1** | | | | | **EA-PP2** | | | |
| Stage 1 | 3000 | 1 HGA, 1 HGB | 10-15% | 7 | 1100 | 1 HGA, 1 HGB | 15-18% | 9 |
| Stage 2 | 900-1050 | 3 HGA, 3 HGB | 15% | 14 | 495-600 | 2 HGA, 2 HGB | 17-20% | 14 |
| Stage 3 | 200-225 | 5 HGA, 5 HGB | *20%* | 17 | 150-200 | 4 HGA, 4 HGB | *15-18%* | 19 |
| Stage 4 | 40-45 | NA | *20%* | 37 | 30-35 | NA | *15-18%* | 34 |
| Stage 5 | 6-10 | NA | *(3-4) ^a^* | 30-50 | 3-5 | NA | *(3-4) ^a^* | 30-40 |
| **EA-PP3** | | | | | **SA-PP1** | | | |
| Stage 1 | 700 | 1 HGA, 1 HGB | 10-15% | 7 | 1800 | 1 HGA, 1 HGB | 10 to 15% | 5 |
| Stage 2 | 210-315 | 3 HGA, 3 HGB | 25-30% | 8 | 800 | 3 HGA, 3 HGB | 15% | 10 |
| Stage 3 | 110-130 | 5 HGA, 5 HGB | *25-30%* | 13 | 220 | 5 HGA, 5 HGB | *20%* | 15 |
| Stage 4 | 20 | NA | *30%* | 17 | 30-40 | NA | *20%* | 30-40 |
| Stage 5 | 2-4 | NA | *(2-3) ^a^* | 15-20 | 6-8 | NA | *(3-4) ^a^* | 60-80 |
| **SA-PP2** | | | | | **SAHDT** | | | |
| Stage 1 | 1000 | 1 HGA, 1 HGB) | 10% | 5 | 1700 | 1 HGA, 1 HGB | 10-12% | 10 |
| Stage 2 | 300 | 2 HGA, 2 HGB | 15% | 10 | 400-600 | 3 HGA, 3 HGB | 15-17% | 13 |
| Stage 3 | 100 | 3 HGA, 3 HGB | *20%* | 15 | 120-160 | 4 HGA, 4 HGB | *18-22%* | 15 |
| Stage 4 | 30 | NA | *20%* | 20-25 | 25-30 | NA | *27-33%* | 15 |
| Stage 5 | 5 | NA | *(2-3) ^a^* | 60 | 10 | NA | *(3) ^a^* | 50-70 |
| **SAWLDT** | | | | | **SADT** | | | |
| Stage 1 | 1250 | 1 HGA, 1 HGB | 8-12% | 9 | 750 | 1 HGA, 1 HGB | 8-12% | 9 |
| Stage 2 | 300-400 | 3 HGA, 3 HGB | 10-15% | 5 | 250-300 | 3 HGA, 3 HGB | 13-16% | 5 |
| Stage 3 | 80-100 | 4 HGA, 4 HGB | *16-20%* | 11 | 50-80 | 4 HGA, 4 HGB | *16-20%* | 8 |
| Stage 4 | 16-20 | NA | *30-33%* | 10 | 15-20 | NA | *30-33%* | 9 |
| Stage 5 | 6-10 | NA | *(2-3) ^a^* | 50-70 | 5-7 | NA | *(1-2) ^a^* | 30-50 |

^a^Number of hybrids selected for release to seed companies; HGA – Heterotic Group A; HGB – Heterotic Group B, NA – Not applicable

**Table S3.** Number of locations used in the estimation of genetic trend for each treatment by breeding pipeline.

| **Region** |  | **Breeding pipeline** | **Treatment** | **2009** | **2010** | **2011** | **2012** | **2013** | **2014** | **2015** | **2016** | **2017** | **2018** | **2019** | **2020** |
| --- | --- | --- | --- | --- | --- | --- | --- | --- | --- | --- | --- | --- | --- | --- | --- |
| Latin America |  | LatAmTL (White) | Rainfed |  |  | 19 | 40 | 49 | 30 | 36 | 52 | 39 | 43 | 44 | 25 |
|  |  | LatAmTL (Yellow) | Rainfed |  |  | 19 | 33 | 47 | 30 | 34 | 51 | 39 | 41 | 44 | 25 |
|  |  | LatAmTM (White) | Rainfed |  |  | 24 | 29 | 30 | 41 | 38 | 42 | 42 | 40 | 45 | 32 |
|  |  | LatAmTM (Yellow) | Rainfed |  |  | 21 | 29 | 30 | 41 | 36 | 41 | 42 | 39 | 31 | 23 |
| Eastern Africa |  | EA-PP1a | Opt |  |  | 10 | 16 | 16 | 22 | 32 | 28 | 22 | 4 | 12 | 7 |
|  |  |  | Drt |  |  | 6 | 4 | 7 | 3 | 6 | 6 | 6 | 2 | 4 | 4 |
|  |  | EA-PP1b | Opt |  |  | 9 | 13 | 15 | 22 | 28 | 30 | 23 | 12 | 6 | 11 |
|  |  |  | Drt |  |  | 6 | 7 | 11 | 7 | 6 | 5 | 7 | 2 | 4 | 5 |
|  |  | EA-PP2 | Opt |  |  | 11 | 17 | 8 | 4 | 4 | 10 | 9 | 9 | 10 |  |
|  |  |  | Drt |  |  | 1 | 2 | 1 | 1 | 1 |  |  | 1 |  |  |
|  |  |  | LN |  |  | 2 | 2 |  | 1 | 4 | 1 | 2 | 4 | 2 |  |
|  |  |  | RS |  |  | 1 | 5 | 1 |  |  |  |  |  | 1 |  |
|  |  | EA-PP3 | Opt |  |  | 4 | 2 | 4 | 9 | 3 | 3 | 5 | 10 | 9 | 11 |
| Southern Africa |  | SA-PP1 | Opt |  |  |  |  | 15 | 25 | 12 | 19 | 22 | 30 | 21 | 17 |
|  |  |  | Drt |  |  |  |  |  | 2 |  |  | 6 | 1 | 4 | 2 |
|  |  |  | LN |  |  |  |  | 5 | 2 | 2 | 9 | 8 | 9 | 8 | 7 |
|  |  |  | RS |  |  |  |  | 18 | 14 | 4 | 9 | 8 | 11 | 8 | 12 |
|  |  | SA-PP2 | Opt |  |  |  |  | 15 | 23 | 29 | 24 | 28 | 23 | 22 | 14 |
|  |  |  | Drt |  |  |  |  | 4 | 2 | 4 | 3 | 7 | 2 | 2 | 5 |
|  |  |  | LN |  |  |  |  | 6 | 5 | 7 | 9 | 7 | 6 | 7 | 4 |
|  |  |  | RS |  |  |  |  | 5 | 15 | 7 | 13 | 2 | 9 | 8 | 8 |
| South Asia |  | SAHDT | LVPD |  | 1 |  |  | 1 | 19 | 32 | 62 | 56 | 7 | 39 |  |
|  |  |  | MVPD | 1 | 1 |  | 1 | 3 | 41 | 43 | 76 | 55 | 31 | 45 | 8 |
|  |  |  | HVPD |  | 3 | 4 | 6 | 15 | 39 | 57 | 90 | 71 | 26 | 38 | 3 |
|  |  | SAWLDT |  |  |  |  | 5 |  | 21 | 13 | 7 | 11 | 10 |  |  |
|  |  | SADT | LYP | 1 |  | 1 | 12 | 7 | 23 | 36 | 13 | 25 | 19 |  | 8 |
|  |  |  | HYP |  |  | 2 | 2 | 1 | 27 | 26 | 17 | 17 | 17 | 17 | 17 |

Opt – Optimal; Drt – Drought; LN – Low N; RS – Random Stress; LVPD – Low VPD; MVPD – Moderate VPD; HVPD – High VPD; LYP – Low Yield Potential;

HYP – High Yield Potential

**Table S4.** Number of genotypes used in the estimation of genetic trend for each breeding pipeline.

| **Region** | **Breeding pipeline** | **2009** | | **2010** | | **2011** | | **2012** | | **2013** | | **2014** | | **2015** | | **2016** | | **2017** | | **2018** | | **2019** | | **2020** | |  |
| --- | --- | --- | --- | --- | --- | --- | --- | --- | --- | --- | --- | --- | --- | --- | --- | --- | --- | --- | --- | --- | --- | --- | --- | --- | --- | --- |
| Latin America | LatAmTL (White) | |  | |  | | 24 | | 32 | | 36 | | 36 | | 21 | | 21 | | 21 | | 27 | | 21 | | 25 | |
|  | LatAmTL (Yellow) | |  | |  | | 9 | | 12 | | 15 | | 12 | | 10 | | 16 | | 12 | | 14 | | 18 | | 18 | |
|  | LatAmTM (White) |  | |  | | 16 | | 15 | | 16 | | 24 | | 18 | | 21 | | 18 | | 18 | | 18 | | 14 | |  |
|  | LatAmTM (Yellow) |  | |  | | 15 | | 7 | | 16 | | 18 | | 18 | | 24 | | 24 | | 30 | | 18 | | 18 | |  |
| Eastern Africa | EA-PP1a |  | |  | | 48 | | 48 | | 48 | | 48 | | 58 | | 59 | | 64 | | 81 | | 59 | | 57 | |  |
|  | EA-PP1b |  | |  | | 50 | | 30 | | 34 | | 34 | | 54 | | 54 | | 54 | | 75 | | 44 | | 41 | |  |
|  | EA-PP2 |  | |  | | 39 | | 39 | | 24 | | 48 | | 59 | | 48 | | 61 | | 61 | | 47 | |  | |  |
|  | EA-PP3 |  | |  | | 6 | | 8 | | 8 | | 19 | | 12 | | 12 | | 11 | | 26 | | 30 | | 29 | |  |
| Southern Africa | SA-PP1 |  | |  | |  | |  | | 62 | | 38 | | 58 | | 62 | | 48 | | 40 | | 40 | | 39 | |  |
|  | SA-PP2 |  | |  | |  | |  | | 49 | | 60 | | 55 | | 55 | | 50 | | 45 | | 40 | | 35 | |  |
| South Asia | SAWLDT |  | |  | |  | | 370 | |  | | 612 | | 88 | | 452 | | 400 | | 399 | |  | |  | |  |
|  | SAHDT (low VPD) |  | | 60 | |  | |  | | 278 | | 529 | | 529 | | 968 | | 194 | | 313 | | 681 | |  | |  |
|  | SAHDT (medium VPD) | 53 | | 101 | |  | | 151 | | 394 | | 1103 | | 938 | | 1093 | | 210 | | 1724 | | 895 | | 745 | |  |
|  | SAHDT (high VPD) |  | | 126 | | 230 | | 513 | | 744 | | 1139 | | 428 | | 1096 | | 208 | | 1075 | | 1331 | | 135 | |  |
|  | SADT (rainfed; low yield) | 171 | |  | | 114 | | 995 | | 1965 | | 1260 | | 612 | | 544 | | 641 | | 391 | |  | | 192 | |  |
|  | SADT (rainfed; high yield) |  | |  | | 252 | | 332 | | 294 | | 630 | | 538 | | 473 | | 699 | | 518 | | 444 | | 274 | |  |

**Table S5.** Description of treatments and the corresponding breeding pipelines.

| **Treatment** | **Description** | **Breeding pipelines** |
| --- | --- | --- |
| Optimal | Conducted in the main season, with optimal levels of fertilization. Supplemental irrigation was applied when required. | EA-PP1, EA-PP2, EA-PP3, SA-PP1, SA-PP2, |
| Rainfed | Conducted in the main season, with optimal levels of fertilization, but with random and varying levels of moisture stress ranging from drought stress to excess moisture. | LatAmTL (White, Yellow) and LatAmTM (White, Yellow) |
| Managed drought stress | Conducted in the off season. Irrigation is withheld approximately 2-3 weeks prior to flowering to allow drought stress at flowering. | EA-PP1, EA-PP2, SA-PP1, SA-PP2 |
| Random stress | Conducted in the main season in drought prone maize growing regions. Other management practices including fertilizer use and agronomy may also be sub-optimal. | EA-PP1, EA-PP2, SA-PP1, SA-PP2 |
| Low nitrogen stress | Experiments were planted on sites that had been depleted of nitrogen for at least four seasons by no nitrogen fertilizer to plots and removing stover from the field after harvest. | EA-PP1, EA-PP2, SA-PP1, SA-PP2 |
| Low, medium, and high vapour pressure deficit (VPD) heat stress | Heat stress trials were conducted under natural heat stress conditions (based historical climate data), where most part of the season is rain-free and there is a window of at least one month when both maximum (T_max_) and minimum temperature (T_min_) is above threshold limits, i.e., T_max_ above 33 and T_min_ above 23^○^C, respectively. For example – in South Asia delayed planting in Spring season (i.e. in mid-March) was identified as most suitable time for planting heat stress trials, where most of part late vegetative stage was exposed to T_max_ >33^○^C during April month and almost all of reproductive stage, including flowering and early grain filling stage was exposed to T_max_ >40^○^C during first fortnight of May month at all the selected locations in heat stress phenotyping network. Also, T_min_ was above >23^○^C at most of the locations during late vegetative stage and at all the phenotyping locations during reproductive stage. However, as heat stress is function of VPD at T_max_, the phenotyping locations were grouped into three types of heat stress sites, including high VPD sites, moderate VPD sites and low VPD sites, where average VPD was >5.0 kPa, 3.0-5.0 kPa and <3.0 kPa, respectively, at T_max_ >35^○^C^45^. | SAHDT |
| Waterlogging stress | Trials were conducted during rainy season and fields inundated with water continuously for seven days at V5-V6 growth stage to mimic the intermittent waterlogging conditions prevailing in the target population of environments (TPE)^46^. | SAWLDT |
| Rainfed low yield (<6 t ha^-1^); Rainfed high yield (>6 t ha^-1^) | Rainfed trials were conducted in the main (rainy) season. The trialling sites are grouped as (i) regions with optimal conditions where in general the maize yield levels are high with best management practices; and (ii) rainfed regions with sub-par management; the yield levels in such environments are generally lower (<6 t/ha) as the crop often faces multitudes of stresses. | SADT |

**Table S6.** Number of years of trials used in the estimation of genetic trend for grain yield

for each breeding pipeline.

| **Region** | **Breeding pipeline** | **Management** | **Years** |
| --- | --- | --- | --- |
| Latin America | LatAmTL (White) | Rainfed | 10 |
|  | LatAmTL (Yellow) | Rainfed | 10 |
|  | LatAmTM (White) | Rainfed | 10 |
|  | LatAmTM (Yellow) | Rainfed | 10 |
| Eastern Africa | EA-PP1a | Optimal | 10 |
|  |  | Managed drought | 10 |
|  | EA-PP1b | Optimal | 10 |
|  |  | Managed drought | 10 |
|  | EA-PP2 | Optimal | 10 |
|  |  | Managed drought | 8 |
|  |  | Low nitrogen | 9 |
|  |  | Random stress | 5 |
|  | EA-PP3 | Optimal | 10 |
| Southern Africa | SA-PP1 | Optimal | 8 |
|  |  | Managed drought | 5 |
|  |  | Low nitrogen | 8 |
|  |  | Random stress | 8 |
|  | SA-PP2 | Optimal | 8 |
|  |  | Managed drought | 8 |
|  |  | Low nitrogen | 8 |
|  |  | Random stress | 8 |
| South Asia | SAHDT | Low VPD | 8 |
|  |  | Medium VPD | 11 |
|  |  | High VPD | 11 |
|  | SAWLDT | Waterlogging | 6 |
|  | SADT | Rainfed (Low yield potential) | 10 |
|  |  | Rainfed (High yield potential) | 10 |

**Table S7.** Initial grain yield at the start of the genetic trend estimation and genetic trends for grain

yield in each of the tropical maize breeding pipelines.

|  |  |  | **Initial grain yield (t ha^-1^)** | **Genetic trend*** | |
| --- | --- | --- | --- | --- | --- |
| **Region** | **Breeding pipeline** | **Management** |  | **kg ha^-1^ yr^-1^** | **% yr^-1^** |
| Latin America | LatAmTL (White) | Rainfed | 7.62 | 66 (21) | 0.87 |
|  | LatAmTL (Yellow) | Rainfed | 7.04 | 143 (44) | 2.04 |
|  | LatAmTM (White) | Rainfed | 6.07 | 117 (15) | 1.93 |
|  | LatAmTM (Yellow) | Rainfed | 5.71 | 122 (18) | 2.14 |
| Eastern Africa | EA-PP1a | Optimal | 5.89 | 88 (13) | 1.50 |
|  | EA-PP1a | Managed drought | 2.61 | 64 (7) | 2.46 |
|  | EA-PP1b | Optimal | 6.60 | NS | NS |
|  | EA-PP1b | Managed drought | 2.65 | 54 (10) | 2.06 |
|  | EA-PP2 | Optimal | 6.23 | 108 (15) | 1.74 |
|  | EA-PP2 | Managed drought | 2.76 | -40 (16) | -1.43 |
|  | EA-PP2 | Low nitrogen | 3.56 | NS | NS |
|  | EA-PP2 | Random stress | 2.66 | 62 (13) | 2.35 |
|  | EA-PP3 | Optimal | 7.26 | NS | Ns |
| Southern Africa | SA-PP1 | Optimal | 7.40 | 80 (22) | 1.08 |
|  | SA-PP1 | Managed drought | 2.15 | NS | NS |
|  | SA-PP1 | Low nitrogen | 3.30 | NS | NS |
|  | SA-PP1 | Random stress | 3.94 | NS | NS |
|  | SA-PP2 | Optimal | 6.91 | 138 (19) | 1.99 |
|  | SA-PP2 | Managed drought | 2.09 | 45 (10) | 2.13 |
|  | SA-PP2 | Low nitrogen | 2.68 | NS | NS |
|  | SA-PP2 | Random stress | 3.77 | 108 (15) | 2.87 |
| South Asia | SAHDT | Low VPD | 5.83 | 118 (12) | 2.02 |
|  | SAHDT | Medium VPD | 4.38 | 36 (7) | 0.82 |
|  | SAHDT | High VPD | 2.68 | NS | NS |
|  | SAWLDT | Waterlogging | 2.48 | 84 (10) | 3.39 |
|  | SADT | Rainfed (Low yield potential) | 4.47 | 71 (10) | 1.59 |
|  | SADT | Rainfed (High yield potential) | 6.78 | NS | NS |

*: Values in parentheses correspond to the standard error of the genetic gain estimation.

NS: Non-significant

| **Table S8**. Number of genotypes per year, and number of genotypes which link pairs of years in different breeding pipelines. | | | | | | | | | | | | | |
| --- | --- | --- | --- | --- | --- | --- | --- | --- | --- | --- | --- | --- | --- |
|  |  |  |  |  |  |  |  |  |  |  |  |  |  |
| **EA-PP1a** | **2011** | **2012** | **2013** | **2014** | **2015** | **2016** | **2017** | **2018** | **2019** | **2020** |  |  |  |
| 2011 | 48 |  |  |  |  |  |  |  |  |  |  |  |  |
| 2012 | 1 | 48 |  |  |  |  |  |  |  |  |  |  |  |
| 2013 | 2 | 7 | 48 |  |  |  |  |  |  |  |  |  |  |
| 2014 | 3 | 2 | 3 | 48 |  |  |  |  |  |  |  |  |  |
| 2015 | 1 | 1 | 2 | 2 | 58 |  |  |  |  |  |  |  |  |
| 2016 | 1 | 0 | 0 | 2 | 2 | 59 |  |  |  |  |  |  |  |
| 2017 | 1 | 0 | 0 | 2 | 2 | 5 | 64 |  |  |  |  |  |  |
| 2018 | 0 | 1 | 1 | 2 | 2 | 1 | 2 | 81 |  |  |  |  |  |
| 2019 | 0 | 0 | 0 | 1 | 0 | 2 | 3 | 3 | 59 |  |  |  |  |
| 2020 | 0 | 0 | 0 | 1 | 1 | 2 | 3 | 6 | 5 | 57 |  |  |  |
|  |  |  |  |  |  |  |  |  |  |  |  |  |  |
| **EA-PP1b** | **2011** | **2012** | **2013** | **2014** | **2015** | **2016** | **2017** | **2018** | **2019** | **2020** |  |  |  |
| 2011 | 50 |  |  |  |  |  |  |  |  |  |  |  |  |
| 2012 | 1 | 30 |  |  |  |  |  |  |  |  |  |  |  |
| 2013 | 1 | 9 | 34 |  |  |  |  |  |  |  |  |  |  |
| 2014 | 1 | 3 | 3 | 34 |  |  |  |  |  |  |  |  |  |
| 2015 | 1 | 3 | 3 | 3 | 54 |  |  |  |  |  |  |  |  |
| 2016 | 1 | 3 | 3 | 3 | 3 | 54 |  |  |  |  |  |  |  |
| 2017 | 1 | 3 | 3 | 3 | 3 | 4 | 54 |  |  |  |  |  |  |
| 2018 | 1 | 3 | 3 | 3 | 3 | 4 | 4 | 75 |  |  |  |  |  |
| 2019 | 1 | 2 | 2 | 2 | 2 | 2 | 2 | 3 | 44 |  |  |  |  |
| 2020 | 1 | 2 | 2 | 2 | 2 | 3 | 3 | 4 | 4 | 41 |  |  |  |
|  |  |  |  |  |  |  |  |  |  |  |  |  |  |
| **EA-PP2** | **2011** | **2012** | **2013** | **2014** | **2015** | **2016** | **2017** | **2018** | **2019** |  |  |  |  |
| 2011 | 39 |  |  |  |  |  |  |  |  |  |  |  |  |
| 2012 | 21 | 39 |  |  |  |  |  |  |  |  |  |  |  |
| 2013 | 3 | 8 | 24 |  |  |  |  |  |  |  |  |  |  |
| 2014 | 2 | 3 | 2 | 48 |  |  |  |  |  |  |  |  |  |
| 2015 | 1 | 1 | 1 | 1 | 59 |  |  |  |  |  |  |  |  |
| 2016 | 3 | 7 | 4 | 2 | 4 | 48 |  |  |  |  |  |  |  |
| 2017 | 1 | 1 | 0 | 1 | 2 | 5 | 61 |  |  |  |  |  |  |
| 2018 | 1 | 1 | 1 | 1 | 4 | 6 | 6 | 61 |  |  |  |  |  |
| 2019 | 1 | 1 | 1 | 0 | 3 | 3 | 1 | 5 | 47 |  |  |  |  |
|  |  |  |  |  |  |  |  |  |  |  |  |  |  |
| **EA-PP3-Highland** | **2011** | **2012** | **2013** | **2014** | **2015** | **2016** | **2017** | **2018** | **2019** | **2020** |  |  |  |
| 2011 | 6 |  |  |  |  |  |  |  |  |  |  |  |  |
| 2012 | 3 | 8 |  |  |  |  |  |  |  |  |  |  |  |
| 2013 | 2 | 2 | 8 |  |  |  |  |  |  |  |  |  |  |
| 2014 | 1 | 2 | 1 | 19 |  |  |  |  |  |  |  |  |  |
| 2015 | 2 | 2 | 2 | 3 | 12 |  |  |  |  |  |  |  |  |
| 2016 | 2 | 2 | 2 | 1 | 2 | 12 |  |  |  |  |  |  |  |
| 2017 | 2 | 2 | 2 | 1 | 2 | 2 | 11 |  |  |  |  |  |  |
| 2018 | 1 | 1 | 1 | 1 | 1 | 1 | 2 | 26 |  |  |  |  |  |
| 2019 | 0 | 0 | 0 | 0 | 0 | 2 | 1 | 1 | 30 |  |  |  |  |
| 2020 | 0 | 0 | 0 | 0 | 0 | 2 | 1 | 2 | 27 | 29 |  |  |  |
|  |  |  |  |  |  |  |  |  |  |  |  |  |  |
| **SA-PP1** | **2013** | **2014** | **2015** | **2016** | **2017** | **2018** | **2019** | **2020** |  |  |  |  |  |
| 2013 | 63 |  |  |  |  |  |  |  |  |  |  |  |  |
| 2014 | 0 | 38 |  |  |  |  |  |  |  |  |  |  |  |
| 2015 | 4 | 0 | 58 |  |  |  |  |  |  |  |  |  |  |
| 2016 | 3 | 0 | 5 | 62 |  |  |  |  |  |  |  |  |  |
| 2017 | 2 | 1 | 2 | 2 | 48 |  |  |  |  |  |  |  |  |
| 2018 | 3 | 1 | 3 | 3 | 19 | 40 |  |  |  |  |  |  |  |
| 2019 | 3 | 1 | 3 | 3 | 9 | 15 | 40 |  |  |  |  |  |  |
| 2020 | 2 | 3 | 3 | 3 | 4 | 5 | 5 | 39 |  |  |  |  |  |
|  |  |  |  |  |  |  |  |  |  |  |  |  |  |
| **SA-PP2** | **2013** | **2014** | **2015** | **2016** | **2017** | **2018** | **2019** | **2020** |  |  |  |  |  |
| 2013 | 49 |  |  |  |  |  |  |  |  |  |  |  |  |
| 2014 | 19 | 60 |  |  |  |  |  |  |  |  |  |  |  |
| 2015 | 9 | 19 | 55 |  |  |  |  |  |  |  |  |  |  |
| 2016 | 4 | 9 | 15 | 55 |  |  |  |  |  |  |  |  |  |
| 2017 | 7 | 6 | 8 | 11 | 50 |  |  |  |  |  |  |  |  |
| 2018 | 6 | 6 | 8 | 10 | 29 | 45 |  |  |  |  |  |  |  |
| 2019 | 6 | 6 | 8 | 9 | 19 | 22 | 40 |  |  |  |  |  |  |
| 2020 | 5 | 5 | 6 | 5 | 9 | 12 | 7 | 35 |  |  |  |  |  |
|  |  |  |  |  |  |  |  |  |  |  |  |  |  |
| **LA-Stage 5-Subtropical-Yellow** | **2011** | **2012** | **2013** | **2014** | **2015** | **2016** | **2017** | **2018** | **2019** | **2020** |  |  |  |
| 2011 | 9 |  |  |  |  |  |  |  |  |  |  |  |  |
| 2012 | 5 | 12 |  |  |  |  |  |  |  |  |  |  |  |
| 2013 | 5 | 7 | 15 |  |  |  |  |  |  |  |  |  |  |
| 2014 | 1 | 1 | 1 | 12 |  |  |  |  |  |  |  |  |  |
| 2015 | 1 | 1 | 1 | 2 | 10 |  |  |  |  |  |  |  |  |
| 2016 | 1 | 2 | 1 | 1 | 2 | 16 |  |  |  |  |  |  |  |
| 2017 | 2 | 2 | 2 | 1 | 1 | 2 | 12 |  |  |  |  |  |  |
| 2018 | 0 | 0 | 0 | 0 | 0 | 0 | 1 | 14 |  |  |  |  |  |
| 2019 | 1 | 1 | 1 | 0 | 0 | 0 | 1 | 1 | 18 |  |  |  |  |
| 2020 | 1 | 1 | 1 | 1 | 1 | 1 | 2 | 1 | 1 | 18 |  |  |  |
|  |  |  |  |  |  |  |  |  |  |  |  |  |  |
| **LA-Stage 5-Subtropical-White** | **2011** | **2012** | **2013** | **2014** | **2015** | **2016** | **2017** | **2018** | **2019** | **2020** |  |  |  |
| 2011 | 24 |  |  |  |  |  |  |  |  |  |  |  |  |
| 2012 | 7 | 32 |  |  |  |  |  |  |  |  |  |  |  |
| 2013 | 7 | 13 | 36 |  |  |  |  |  |  |  |  |  |  |
| 2014 | 3 | 5 | 10 | 36 |  |  |  |  |  |  |  |  |  |
| 2015 | 1 | 2 | 2 | 4 | 21 |  |  |  |  |  |  |  |  |
| 2016 | 0 | 1 | 1 | 3 | 1 | 21 |  |  |  |  |  |  |  |
| 2017 | 3 | 1 | 2 | 3 | 2 | 2 | 21 |  |  |  |  |  |  |
| 2018 | 2 | 1 | 2 | 1 | 1 | 0 | 3 | 27 |  |  |  |  |  |
| 2019 | 0 | 0 | 0 | 0 | 0 | 0 | 2 | 1 | 21 |  |  |  |  |
| 2020 | 0 | 0 | 0 | 0 | 0 | 0 | 3 | 2 | 4 | 25 |  |  |  |
|  |  |  |  |  |  |  |  |  |  |  |  |  |  |
| **LA-Stage 5-Tropical-Yellow** | **2011** | **2012** | **2013** | **2014** | **2015** | **2016** | **2017** | **2018** | **2019** | **2020** |  |  |  |
| 2011 | 15 |  |  |  |  |  |  |  |  |  |  |  |  |
| 2012 | 2 | 7 |  |  |  |  |  |  |  |  |  |  |  |
| 2013 | 0 | 1 | 8 |  |  |  |  |  |  |  |  |  |  |
| 2014 | 0 | 0 | 1 | 9 |  |  |  |  |  |  |  |  |  |
| 2015 | 0 | 1 | 0 | 1 |  |  |  |  |  |  |  |  |  |
| 2016 | 0 | 1 | 0 | 1 | 3 | 12 |  |  |  |  |  |  |  |
| 2017 | 0 | 0 | 0 | 1 | 1 | 3 | 12 |  |  |  |  |  |  |
| 2018 | 0 | 0 | 1 | 1 | 2 | 2 | 3 | 15 |  |  |  |  |  |
| 2019 | 0 | 0 | 0 | 0 | 0 | 1 | 3 | 1 |  |  |  |  |  |
| 2020 | 0 | 0 | 0 | 1 | 1 | 2 | 3 | 1 | 3 | 9 |  |  |  |
|  |  |  |  |  |  |  |  |  |  |  |  |  |  |
| **LA-Stage 5-Tropical-White** | **2011** | **2012** | **2013** | **2014** | **2015** | **2016** | **2017** | **2018** | **2019** | **2020** |  |  |  |
| 2011 | 16 |  |  |  |  |  |  |  |  |  |  |  |  |
| 2012 | 4 | 15 |  |  |  |  |  |  |  |  |  |  |  |
| 2013 | 4 | 4 | 16 |  |  |  |  |  |  |  |  |  |  |
| 2014 | 2 | 2 | 4 | 24 |  |  |  |  |  |  |  |  |  |
| 2015 | 2 | 2 | 3 | 4 | 18 |  |  |  |  |  |  |  |  |
| 2016 | 2 | 1 | 2 | 4 | 3 | 21 |  |  |  |  |  |  |  |
| 2017 | 0 | 0 | 0 | 1 | 2 | 1 | 18 |  |  |  |  |  |  |
| 2018 | 0 | 0 | 0 | 1 | 2 | 1 | 4 | 18 |  |  |  |  |  |
| 2019 | 0 | 0 | 0 | 1 | 2 | 1 | 2 | 4 | 18 |  |  |  |  |
| 2020 | 0 | 0 | 0 | 1 | 1 | 2 | 2 | 2 | 2 | 14 |  |  |  |
|  |  |  |  |  |  |  |  |  |  |  |  |  |  |
| **Asia-SAWLDT** | **2012** | **2014** | **2015** | **2016** | **2017** | **2018** |  |  |  |  |  |  |  |
| 2012 | 370 |  |  |  |  |  |  |  |  |  |  |  |  |
| 2014 | 69 | 612 |  |  |  |  |  |  |  |  |  |  |  |
| 2015 | 2 | 39 | 88 |  |  |  |  |  |  |  |  |  |  |
| 2016 | 35 | 44 | 3 | 452 |  |  |  |  |  |  |  |  |  |
| 2017 | 4 | 9 | 6 | 60 | 400 |  |  |  |  |  |  |  |  |
| 2018 | 2 | 3 | 2 | 18 | 76 | 399 |  |  |  |  |  |  |  |
|  |  |  |  |  |  |  |  |  |  |  |  |  |  |
| **Asia - Spring season, high vapour pressure deficit (VPD) heat, i.e. dry heat, Trial Mean <4 t/ha** | **2010** | **2011** | **2012** | **2013** | **2014** | **2015** | **2016** | **2017** | **2018** | **2019** | **2020** |  |  |
| 2010 | 126 |  |  |  |  |  |  |  |  |  |  |  |  |
| 2011 | 15 | 230 |  |  |  |  |  |  |  |  |  |  |  |
| 2012 | 11 | 0 | 513 |  |  |  |  |  |  |  |  |  |  |
| 2013 | 16 | 2 | 179 | 744 |  |  |  |  |  |  |  |  |  |
| 2014 | 9 | 0 | 167 | 604 | 1139 |  |  |  |  |  |  |  |  |
| 2015 | 5 | 0 | 30 | 76 | 106 | 428 |  |  |  |  |  |  |  |
| 2016 | 0 | 0 | 6 | 25 | 43 | 100 | 1096 |  |  |  |  |  |  |
| 2017 | 1 | 0 | 7 | 14 | 25 | 48 | 92 | 208 |  |  |  |  |  |
| 2018 | 0 | 0 | 3 | 10 | 14 | 20 | 108 | 90 | 1075 |  |  |  |  |
| 2019 | 0 | 0 | 1 | 3 | 5 | 5 | 27 | 37 | 131 | 1331 |  |  |  |
| 2020 | 2 | 0 | 2 | 2 | 2 | 1 | 1 | 2 | 12 | 3 | 135 |  |  |
|  |  |  |  |  |  |  |  |  |  |  |  |  |  |
| **Asia - Spring season, medium vapour pressure deficit (VPD) heat, Trial Mean 4-6 t/ha** | **2009** | **2010** | **2012** | **2013** | **2014** | **2015** | **2016** | **2017** | **2018** | **2019** | **2020** |  |  |
| 2009 | 53 |  |  |  |  |  |  |  |  |  |  |  |  |
| 2010 | 11 | 101 |  |  |  |  |  |  |  |  |  |  |  |
| 2012 | 3 | 4 | 151 |  |  |  |  |  |  |  |  |  |  |
| 2013 | 0 | 6 | 34 | 394 |  |  |  |  |  |  |  |  |  |
| 2014 | 5 | 10 | 100 | 196 | 1103 |  |  |  |  |  |  |  |  |
| 2015 | 3 | 8 | 9 | 25 | 142 | 938 |  |  |  |  |  |  |  |
| 2016 | 1 | 0 | 5 | 10 | 63 | 97 | 1093 |  |  |  |  |  |  |
| 2017 | 0 | 1 | 4 | 5 | 33 | 48 | 93 | 210 |  |  |  |  |  |
| 2018 | 0 | 0 | 2 | 6 | 26 | 21 | 108 | 90 | 1724 |  |  |  |  |
| 2019 | 0 | 0 | 0 | 1 | 6 | 5 | 25 | 37 | 152 | 895 |  |  |  |
| 2020 | 1 | 2 | 2 | 1 | 4 | 0 | 1 | 2 | 21 | 10 | 745 |  |  |
|  |  |  |  |  |  |  |  |  |  |  |  |  |  |
| **Asia - Spring season, low vapour pressure deficit (VPD) heat, i.e. humid heat, >6 t/ha** | **2010** | **2013** | **2014** | **2015** | **2016** | **2017** | **2018** | **2019** |  |  |  |  |  |
| 2010 | 60 |  |  |  |  |  |  |  |  |  |  |  |  |
| 2013 | 0 | 278 |  |  |  |  |  |  |  |  |  |  |  |
| 2014 | 3 | 44 | 529 |  |  |  |  |  |  |  |  |  |  |
| 2015 | 2 | 21 | 66 | 529 |  |  |  |  |  |  |  |  |  |
| 2016 | 0 | 11 | 12 | 80 | 968 |  |  |  |  |  |  |  |  |
| 2017 | 0 | 5 | 13 | 48 | 83 | 194 |  |  |  |  |  |  |  |
| 2018 | 0 | 1 | 7 | 17 | 23 | 61 | 313 |  |  |  |  |  |  |
| 2019 | 0 | 0 | 6 | 5 | 25 | 36 | 46 | 681 |  |  |  |  |  |
|  |  |  |  |  |  |  |  |  |  |  |  |  |  |
| **Dry season managed drought stress** | **2010** | **2011** | **2012** | **2013** | **2014** | **2015** | **2016** | **2017** | **2018** | **2019** | **2020** | **2021** |  |
| 2010 | 242 |  |  |  |  |  |  |  |  |  |  |  |  |
| 2011 | 73 | 1871 |  |  |  |  |  |  |  |  |  |  |  |
| 2012 | 7 | 131 | 2180 |  |  |  |  |  |  |  |  |  |  |
| 2013 | 48 | 210 | 240 | 1945 |  |  |  |  |  |  |  |  |  |
| 2014 | 5 | 28 | 29 | 71 | 506 |  |  |  |  |  |  |  |  |
| 2015 | 5 | 36 | 30 | 79 | 20 | 497 |  |  |  |  |  |  |  |
| 2016 | 0 | 3 | 4 | 4 | 57 | 9 | 346 |  |  |  |  |  |  |
| 2017 | 5 | 42 | 58 | 87 | 39 | 57 | 8 | 1211 |  |  |  |  |  |
| 2018 | 1 | 17 | 18 | 29 | 23 | 22 | 9 | 211 | 901 |  |  |  |  |
| 2019 | 1 | 10 | 10 | 13 | 10 | 14 | 5 | 106 | 219 | 1556 |  |  |  |
| 2020 | 1 | 2 | 1 | 2 | 2 | 3 | 1 | 12 | 6 | 49 | 60 |  |  |
| 2021 | 1 | 4 | 3 | 3 | 3 | 5 | 2 | 27 | 6 | 138 | 26 | 227 |  |
|  |  |  |  |  |  |  |  |  |  |  |  |  |  |
| **Wet season, Rainfed, Optimal, Trial Mean** | **2009** | **2011** | **2012** | **2013** | **2014** | **2015** | **2016** | **2017** | **2018** | **2019** | **2020** |  |  |
| 2009 | 171 |  |  |  |  |  |  |  |  |  |  |  |  |
| 2011 | 0 | 361 |  |  |  |  |  |  |  |  |  |  |  |
| 2012 | 41 | 16 | 1313 |  |  |  |  |  |  |  |  |  |  |
| 2013 | 34 | 4 | 501 | 2238 |  |  |  |  |  |  |  |  |  |
| 2014 | 22 | 5 | 245 | 320 | 1579 |  |  |  |  |  |  |  |  |
| 2015 | 4 | 6 | 72 | 163 | 286 | 888 |  |  |  |  |  |  |  |
| 2016 | 9 | 6 | 76 | 90 | 78 | 50 | 715 |  |  |  |  |  |  |
| 2017 | 8 | 5 | 36 | 63 | 72 | 93 | 157 | 775 |  |  |  |  |  |
| 2018 | 2 | 3 | 34 | 37 | 38 | 28 | 122 | 224 | 735 |  |  |  |  |
| 2019 | 0 | 2 | 5 | 5 | 7 | 5 | 14 | 43 | 140 | 444 |  |  |  |
| 2020 | 0 | 2 | 3 | 5 | 6 | 5 | 10 | 38 | 96 | 111 | 323 |  |  |
|  |  |  |  |  |  |  |  |  |  |  |  |  |  |
| **Wet season, Rainfed, Optimal, Trial Mean <6 t/ha** | **2009** | **2011** | **2012** | **2013** | **2014** | **2015** | **2016** | **2017** | **2018** | **2020** |  |  |  |
| 2009 | 171 |  |  |  |  |  |  |  |  |  |  |  |  |
| 2011 | 0 | 114 |  |  |  |  |  |  |  |  |  |  |  |
| 2012 | 41 | 5 | 995 |  |  |  |  |  |  |  |  |  |  |
| 2013 | 21 | 4 | 325 | 1965 |  |  |  |  |  |  |  |  |  |
| 2014 | 16 | 5 | 154 | 152 | 1260 |  |  |  |  |  |  |  |  |
| 2015 | 2 | 5 | 54 | 55 | 196 | 612 |  |  |  |  |  |  |  |
| 2016 | 9 | 5 | 66 | 63 | 50 | 33 | 544 |  |  |  |  |  |  |
| 2017 | 8 | 4 | 34 | 51 | 49 | 62 | 114 | 641 |  |  |  |  |  |
| 2018 | 2 | 3 | 30 | 25 | 25 | 17 | 59 | 91 |  |  |  |  |  |
| 2020 | 0 | 2 | 3 | 4 | 5 | 3 | 5 | 15 | 43 | 192 |  |  |  |
|  |  |  |  |  |  |  |  |  |  |  |  |  |  |
| **Wet season, Rainfed, Optimal, Trial Mean >6 t/ha** | **2011** | **2012** | **2013** | **2014** | **2015** | **2016** | **2017** | **2018** | **2019** | **2020** |  |  |  |
| 2011 | 252 |  |  |  |  |  |  |  |  |  |  |  |  |
| 2012 | 5 | 332 |  |  |  |  |  |  |  |  |  |  |  |
| 2013 | 3 | 5 | 294 |  |  |  |  |  |  |  |  |  |  |
| 2014 | 2 | 12 | 66 | 630 |  |  |  |  |  |  |  |  |  |
| 2015 | 6 | 9 | 29 | 97 | 538 |  |  |  |  |  |  |  |  |
| 2016 | 6 | 15 | 35 | 56 | 43 | 473 |  |  |  |  |  |  |  |
| 2017 | 5 | 10 | 19 | 37 | 54 | 119 | 699 |  |  |  |  |  |  |
| 2018 | 3 | 6 | 6 | 9 | 14 | 61 | 195 | 518 |  |  |  |  |  |
| 2019 | 2 | 4 | 3 | 3 | 5 | 9 | 43 | 85 | 444 |  |  |  |  |
| 2020 | 2 | 3 | 1 | 2 | 3 | 6 | 36 | 71 | 108 | 274 |  |  |  |
